# Supplementary material for: The impact of site-specific digital histology signatures on deep learning model accuracy and bias
Source: Nat Commun. 2021 Jul 20;12:4423. doi: 10.1038/s41467-021-24698-1 (PMC8292530; doi:10.1038/s41467-021-24698-1)
Supplement: Supplementary file 1 — Supplementary Information [file 41467_2021_24698_MOESM1_ESM.pdf]

The Impact of Site-Specific Digital Histology Signatures on Deep Learning Model Accuracy and Bias

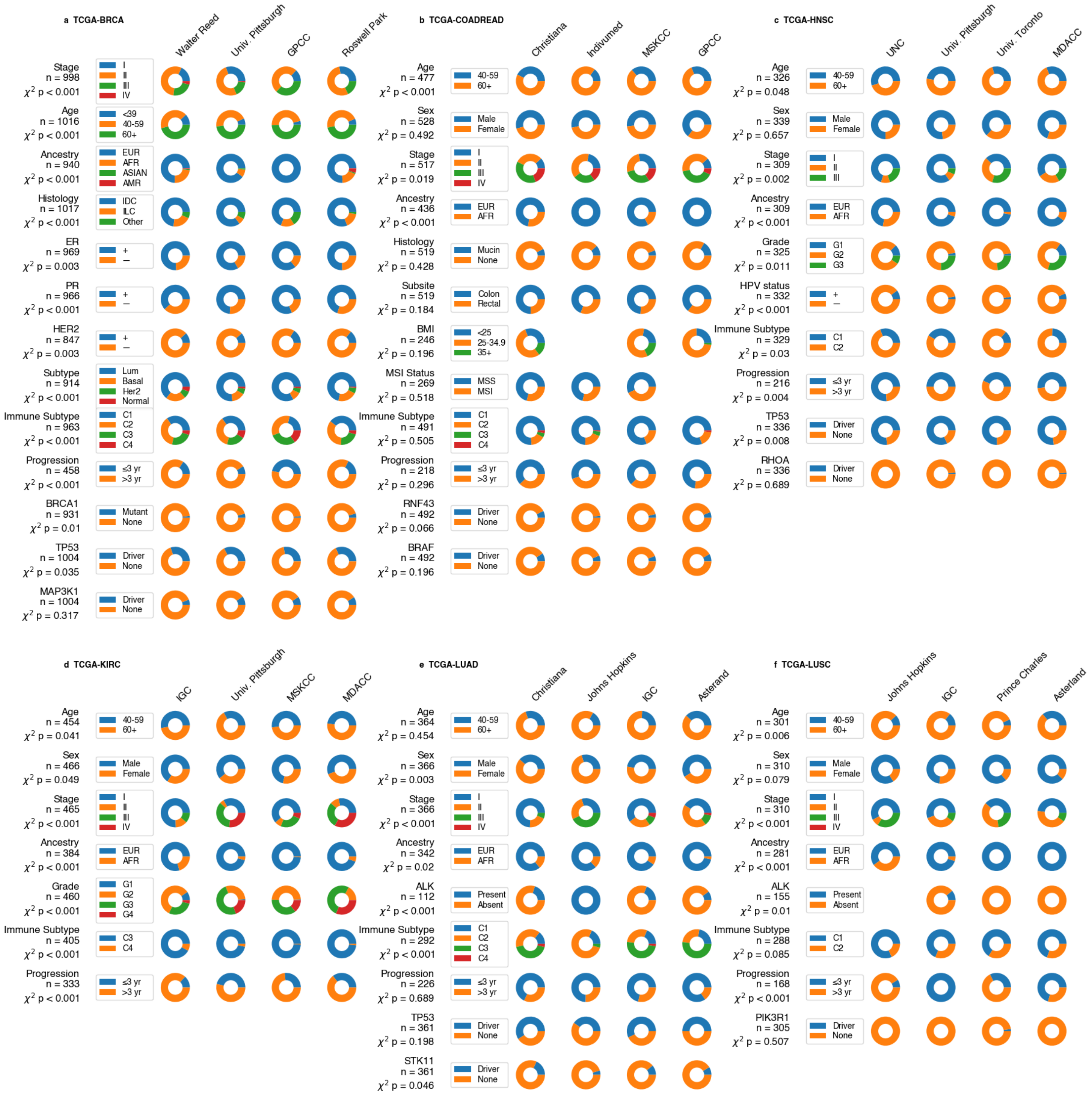

Supplementary Figure 1. Demographics and Tumor Characteristics of Breast Cancer Variability across TCGA. Top four contributing sites are included. Indicated n and χ^2 values listed are for sites contributing over 20 slides (as per Supplementary Table 1). **a.** Breast adenocarcinoma. **b.** Colorectal adenocarcinoma. **c.** Head and neck squamous cell carcinoma. **d.** Clear cell renal carcinoma. **e**. Lung adenocarcinoma. **f.** Lung squamous cell carcinoma. Abbreviations: GPCC = Greater Poland Cancer Center. MSKCC = Memorial Sloan Kettering Cancer Center. UNC = University of North

Carolina. MDACC = MD Anderson Cancer Center. IGC = International Genomic Consortium. EUR = European. AFR = African. AMR = Native American. IDC = Invasive Ductal Carcinoma. ILC = Invasive Lobular Carcinoma. BMI = Body Mass Index. MSI = Microsatellite Instability. MSS = Microsatellite Stable.

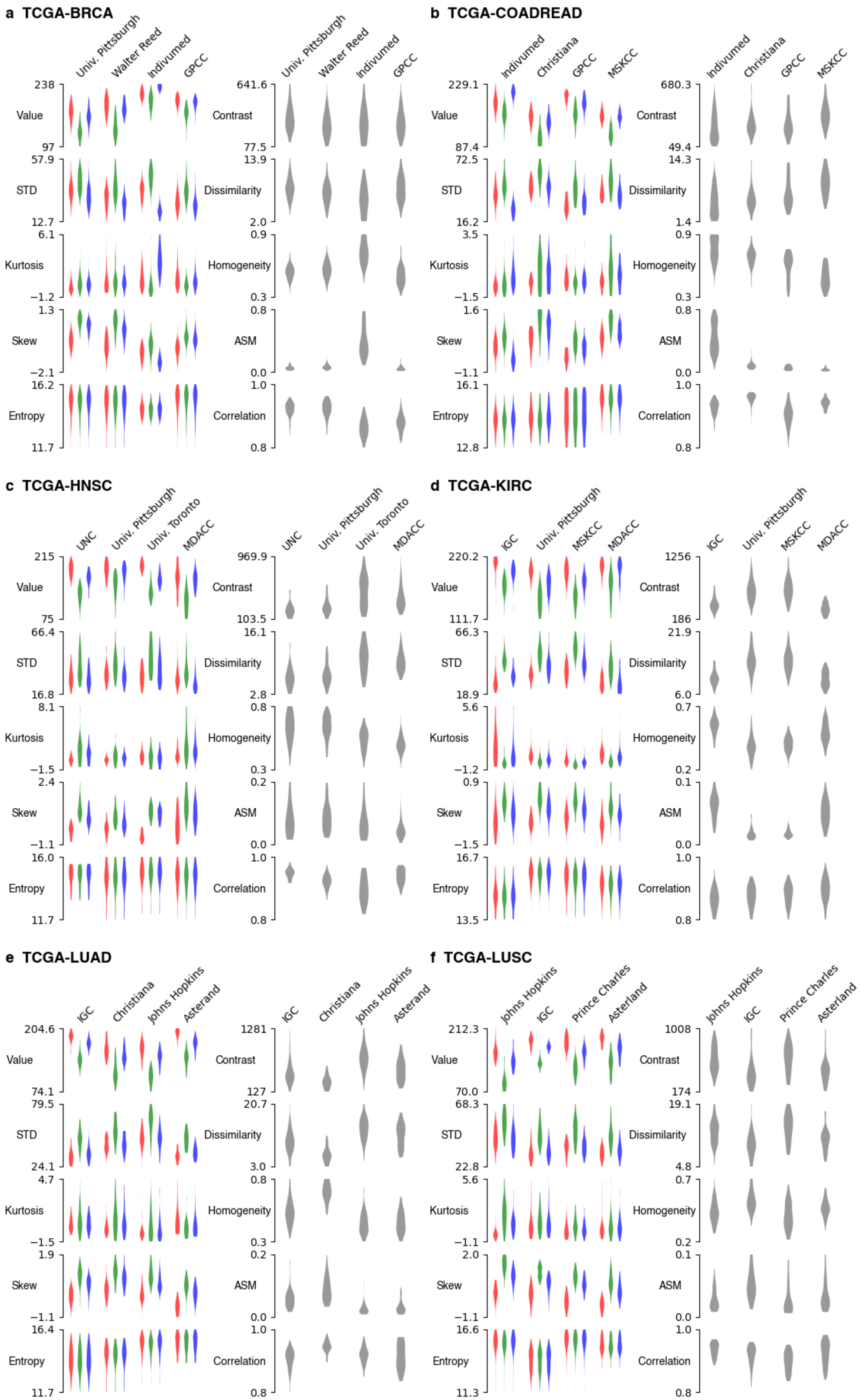

a TCGA-BRCA
Univ. Pittsburgh
Walter Reed
Indivumed
GPCC
Value
238
97
STD
57.9
12.7
Kurtosis
6.1
−1.2
Skew
1.3
−2.1
Entropy
16.2
11.7
Contrast
641.6
77.5
Dissimilarity
13.9
2.0
Homogeneity
0.9
0.3
ASM
0.8
0.0
Correlation
1.0
0.8
b TCGA-COADREAD
Indivumed
Christiana
GPCC
MSKCC
Value
229.1
87.4
STD
72.5
16.2
Kurtosis
3.5
−1.5
Skew
1.6
−1.1
Entropy
16.1
12.8
Contrast
680.3
49.4
Dissimilarity
14.3
1.4
Homogeneity
0.9
0.3
ASM
0.8
0.0
Correlation
1.0
0.8
c TCGA-HNSC
UNC
Univ. Pittsburgh
Univ. Toronto
MDACC
Value
215
75
STD
66.4
16.8
Kurtosis
8.1
−1.5
Skew
2.4
−1.1
Entropy
16.0
11.7
Contrast
969.9
103.5
Dissimilarity
16.1
2.8
Homogeneity
0.8
0.3
ASM
0.2
0.0
Correlation
1.0
0.8
d TCGA-KIRC
IGC
Univ. Pittsburgh
MSKCC
MDACC
Value
220.2
111.7
STD
66.3
18.9
Kurtosis
5.6
−1.2
Skew
0.9
−1.5
Entropy
16.7
13.5
Contrast
1256
186
Dissimilarity
21.9
6.0
Homogeneity
0.7
0.2
ASM
0.1
0.0
Correlation
1.0
0.8
e TCGA-LUAD
IGC
Christiana
Johns Hopkins
Asterand
Value
204.6
74.1
STD
79.5
24.1
Kurtosis
4.7
−1.5
Skew
1.9
−1.1
Entropy
16.4
11.7
Contrast
1281
127
Dissimilarity
20.7
3.0
Homogeneity
0.8
0.3
ASM
0.2
0.0
Correlation
1.0
0.8
f TCGA-LUSC
Johns Hopkins
IGC
Prince Charles
Asterand
Value
212.3
70.0
STD
68.3
22.8
Kurtosis
5.6
−1.1
Skew
2.0
−1.1
Entropy
16.6
11.3
Contrast
1008
174
Dissimilarity
19.1
4.8
Homogeneity
0.7
0.2
ASM
0.1
0.0
Correlation
1.0
0.8

Supplementary Figure 2. Variance in Digital Histology Image Characteristics in TCGA. Top four contributing sites are included. First order slide characteristics are featured in color, and second order characteristics featured in grayscale. **a.** Breast adenocarcinoma (n= 432 slides). **b.** Colorectal adenocarcinoma. (n = 389 slides). **c.** Head and neck squamous cell carcinoma. (n = 228 slides). **d.** Clear cell renal carcinoma. (n = 358 slides). **e**. Lung adenocarcinoma. (n = 255 slides). **f.** Lung squamous cell carcinoma. (n = 182 slides). Abbreviations: STD = Standard Deviation. ASM = Angular Second Moment. GPCC = Greater Poland Cancer Center. MSKCC = Memorial Sloan Kettering Cancer Center. UNC = University of North Carolina. MDACC = MD Anderson Cancer Center. IGC = International Genomic Consortium.

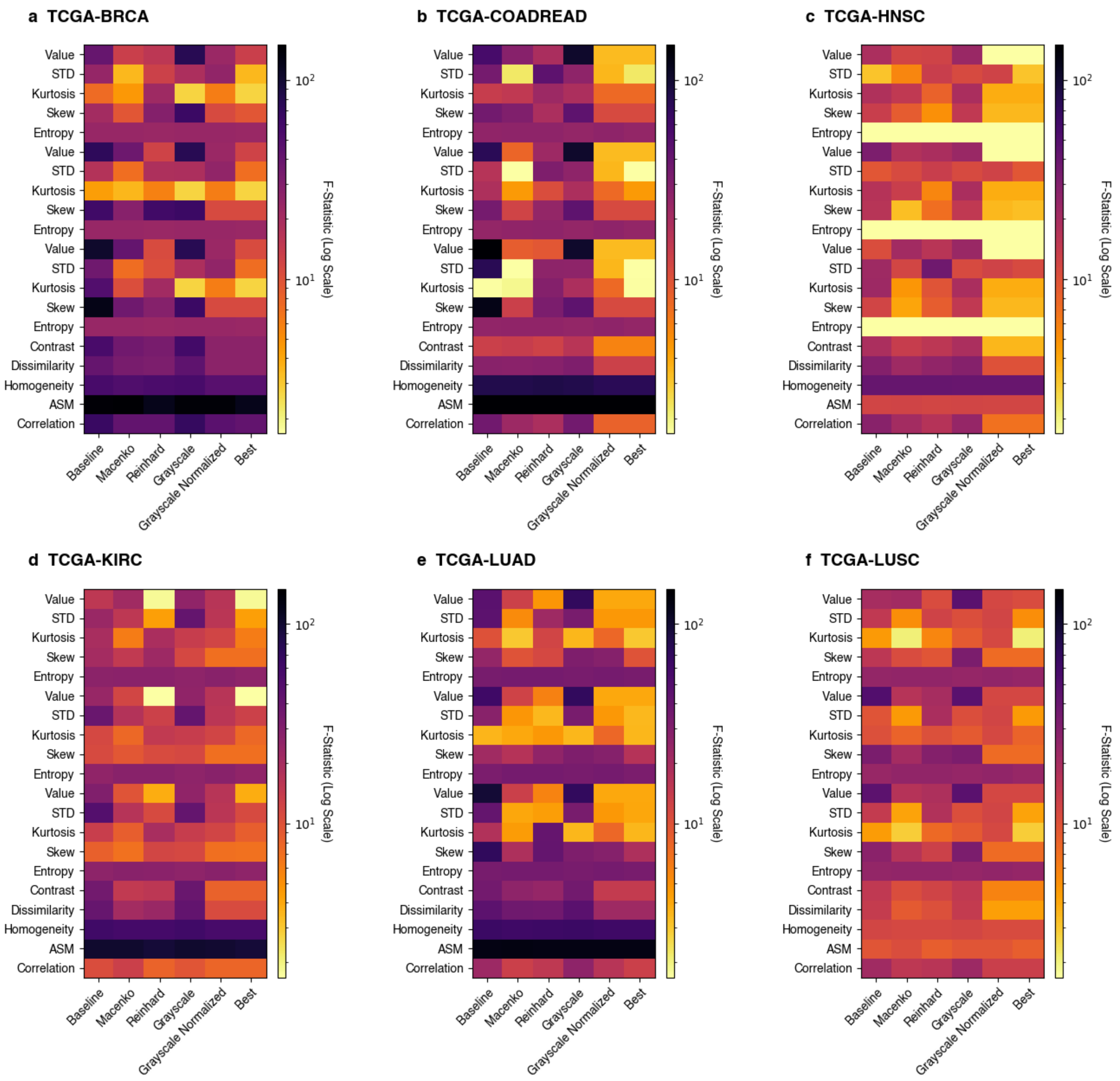

Supplementary Figure 3. ANOVA F-Statistic for First and Second Order Image Features Across TCGA. F-statistic is listed for multiple methods of stain normalization, with the lowest F-statistic (least variability) with any method of normalization indicated in the rightmost column. **a.** Breast adenocarcinoma (n= 888 slides). **b.** Colorectal adenocarcinoma. (n = 495 slides). **c.** Head and neck squamous cell carcinoma. (n = 228 slides). **d.** Clear cell renal carcinoma. (n = 456 slides). **e**. Lung adenocarcinoma. (n = 355 slides). **f.** Lung squamous cell carcinoma. (n = 306 slides). Abbreviations: STD = Standard Deviation. ASM = Angular Second Moment.

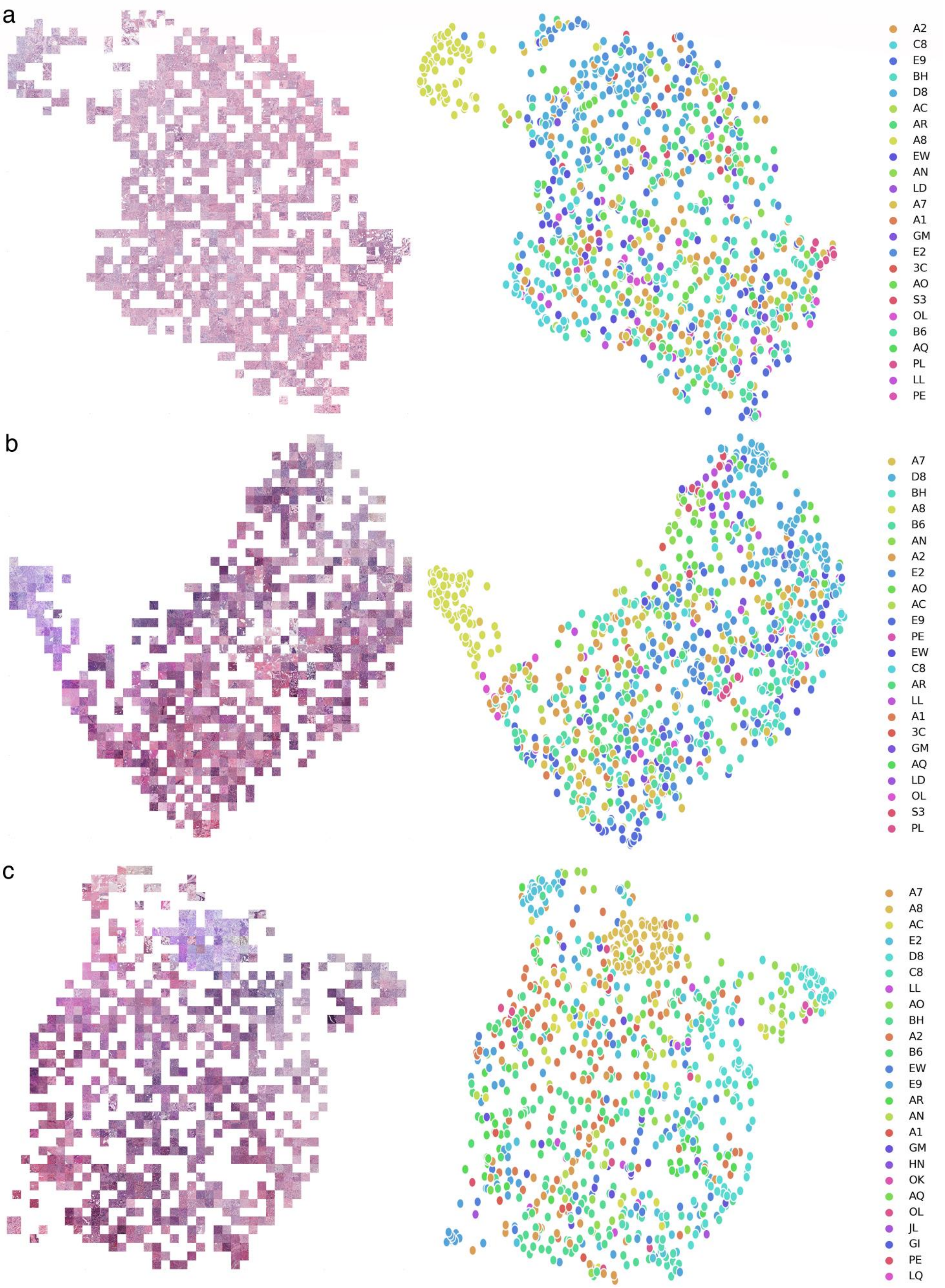
a
A2
C8
E9
BH
D8
AC
AR
A8
EW
AN
LD
A7
A1
GM
E2
3C
AO
S3
OL
B6
AQ
PL
LL
PE
b
A7
D8
BH
A8
B6
AN
A2
E2
AO
AC
E9
PE
EW
C8
AR
LL
A1
3C
GM
AQ
LD
OL
S3
PL
c
A7
A8
AC
E2
D8
C8
LL
AO
BH
A2
B6
EW
E9
AR
AN
A1
GM
HN
OK
AQ
OL
JL
GI
PE
LQ

Supplementary Figure 4. Paired Mosaic and UMAP for Final Layer Activations, Select Models Trained on TCGA-BRCA.

a. Mosaic and UMAP for site detection model, Macenko normalized tiles. **b.** Mosaic and UMAP for ancestry detection, no normalization. **c.** Mosaic and UMAP for BRCA1 detection, no normalization.

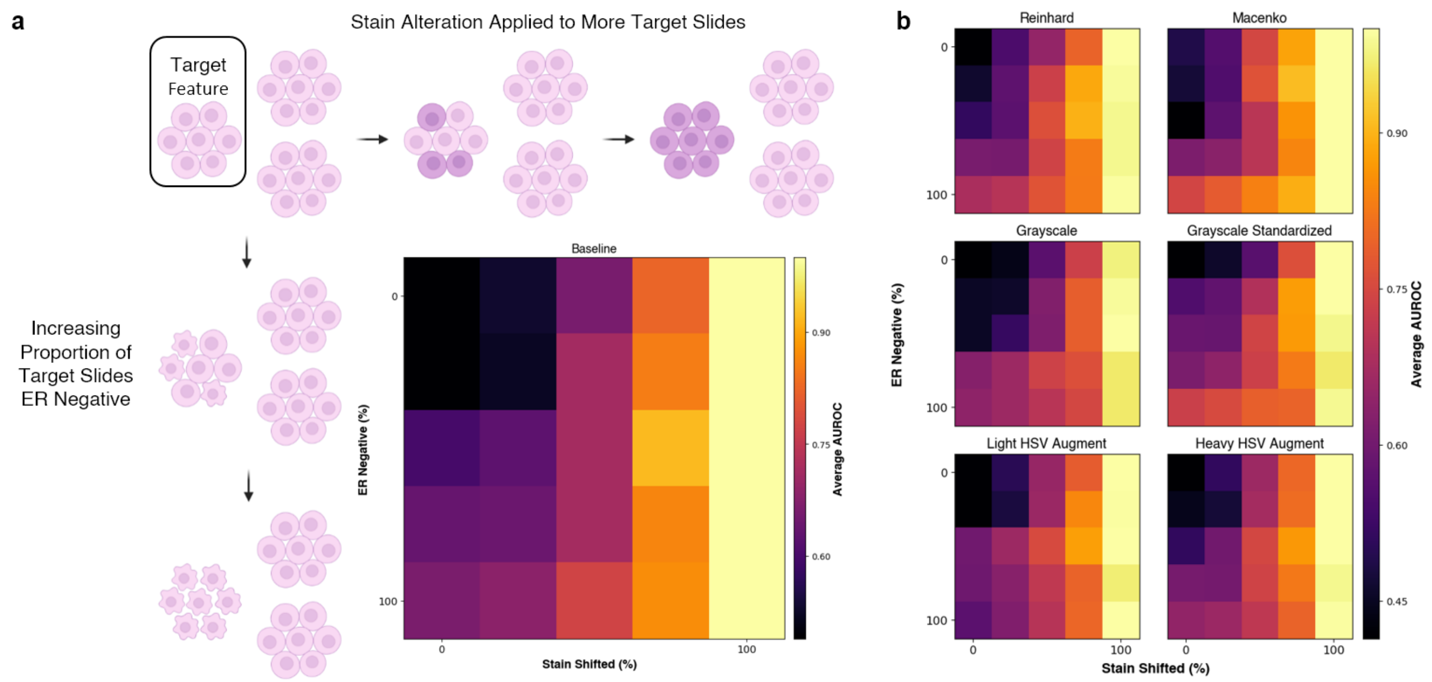

Supplementary Figure 5. Accuracy in Predicting a Synthetic Feature, as a Function of Biologic / Stain related confounders. a. A series of experiments were performed, with models trained to identify a synthetic feature of interest from among n = 69 slides. All slides were chosen from University of Pittsburgh, the largest single site contributing to the TCGA Breast Cancer Cohort, to prevent the influence of external batch effect on experiments – and all slides except the feature of interest were taken from ER positive cancers. Biologic and stain related confounders were introduced – the x-axis signifies the percentage of slides in the target feature set which were ER negative, varying from 0 – 100%. Similarly, the y-axis represents the percentage of slides in the target feature set for which an artificial stain artifact was applied. The stain artifact consisted of an up to 5% shift in the hue, saturation and value of a slide. As expected, as the synthetic feature became increasing ER negative, it was more readily distinguished from the background ER positive slides. However, once artificial stain related batch effect was introduced, model accuracy became less dependent on ER positivity. **b.** This relationship held true for all forms of stain normalization and augmentation, with a slight decrease in maximum AUROC, consistent with results seen for site prediction, suggesting that stain related abnormalities are a predominant source of site-specific histologic signatures in TCGA, and are not fully mitigated with normalization / augmentation. Abbreviations: HSV = Hue Saturation Value. AUROC = Area Under the Receive Operating Characteristic Curve.

Supplementary Table 1. Variance in Demographic and Basic Tumor Characteristics by Site, Select Solid Tumors, The Cancer Genome Atlas. Limited to sites submitting over 20 samples.

Dataset	Variable	# Samples	# Sites	Degrees of Freedom	Chi-squared statistic	Corrected p-value
BRCA	Stage	998	15	42	99.998	3.21×10^{-6} *
	Age	1016	15	28	59.117	8.57×10^{-4} *
	Ancestry	940	15	42	1070.007	9.17×10^{-196} *
	Histology	1017	15	28	128.626	4.92×10^{-14} *
	ER	969	15	14	34.157	2.83×10^{-3} *
	PR	966	15	14	40.047	4.66×10^{-4} *
	HER2	847	15	14	33.757	2.91×10^{-3} *
	BRCA1	931	15	14	29.561	1.04×10^{-2} *
	PAM50 Subtype	914	15	42	111.914	1.24×10^{-7} *
	Immune Subtype	1002	15	56	99.836	4.66×10^{-4} *
	3 Year PFS	458	14	13	58.441	3.24×10^{-7} *
	TP53	1004	15	14	25.242	3.81×10^{-2} *
	MAP3K1	1004	15	14	15.936	3.44×10^{-1}
COAD	Age	477	8	7	32.326	2.12×10^{-4} *
	Sex	528	8	7	7.182	4.92×10^{-1}
	Stage	517	8	21	41.665	1.85×10^{-2} *
	Ancestry	436	8	7	73.734	3.11×10^{-12} *
	Histologic Subtype	519	8	7	8.131	4.28×10^{-1}
	Subsite	519	8	7	12.821	1.84×10^{-1}
	BMI	246	7	12	18.449	1.96×10^{-1}
	MSI Status	269	4	3	2.272	5.18×10^{-1}
	Immune Subtype	491	8	21	20.937	5.05×10^{-1}
	3 Year PFS	218	8	7	9.845	2.96×10^{-1}
	RNF43	492	8	7	16.379	6.56×10^{-2}
	BRAF	492	8	7	11.607	1.96×10^{-1}
HNSC	Age	326	7	6	13.322	4.78×10^{-2} *
	Sex	339	7	6	4.637	6.57×10^{-1}
	Stage	309	7	12	34.929	1.60×10^{-3} *
	Ancestry	309	7	6	35.71	3.14×10^{-5} *
	Grade	325	7	12	27.504	1.09×10^{-2} *
	HPV status	332	7	6	33.617	3.99×10^{-5} *
	Immune Subtype	329	7	6	14.936	2.97×10^{-2} *
	3 Year PFS	216	7	6	21.117	4.37×10^{-3} *
	TP53	336	7	6	19.064	8.11×10^{-3} *
	RHOA	336	7	6	3.913	6.89×10^{-1}
KIRC	Age	454	7	6	13.516	4.15×10^{-2} *
	Sex	466	7	6	12.669	4.86×10^{-2} *
	Stage	465	7	18	109.573	2.65×10^{-14} *
	Ancestry	384	7	6	67.726	4.18×10^{-12} *
	Grade	460	7	18	75.08	1.43×10^{-8} *
	Immune Subtype	405	7	6	23.729	9.97×10^{-4} *
	3 Year PFS	333	7	6	23.267	9.97×10^{-4} *
LUAD	Age	364	8	7	7.245	4.54×10^{-1}

	Sex	366	8	7	23.838	2.74×10^{-3} *
	Stage	366	8	21	89.472	1.79×10^{-9} *
	Ancestry	342	8	7	18.234	1.97×10^{-2} *
	ALK Translocation Status	112	7	6	36.209	7.53×10^{-6} *
	Immune Subtype	292	8	21	80.187	3.38×10^{-8} *
	3 Year PFS	226	8	7	4.759	6.89×10^{-1}
	TP53 Driver	361	8	7	10.661	1.98×10^{-1}
	STK11 Driver	361	8	7	15.474	4.56×10^{-2} *
**LUSC**	Age	301	8	7	21.58	6.00×10^{-3} *
	Sex	310	8	7	13.594	7.85×10^{-2}
	Stage	310	8	21	56.966	9.69×10^{-5} *
	Ancestry	281	8	7	83.467	2.16×10^{-14} *
	ALK Translocation Status	155	6	5	16.328	9.55×10^{-3} *
	Immune Subtype	288	8	7	12.915	8.48×10^{-2}
	3 Year PFS	168	8	7	49.82	6.27×10^{-8} *
	PIK3R1 Driver	305	8	7	6.283	5.07×10^{-1}

(*) indicates p-value remained significant even with Benjamini-Hochberg correction for a false discovery rate of 0.05 (calculated per disease type)

Supplementary Table 2. ANOVA F-Statistic for First and Second Order Image Characteristics, Select Solid Tumors from The Cancer Genome Atlas. Limited to sites submitting over 20 slides.

Dataset	Statistic	Baseline	Macenko	Reinhard	Grayscale	Grayscale Normalized
**BRCA** n = 888 slides, 14 sites	Red	40.4*	13.3*	15.8*	78.6*	23.1*
	Red STD	24.4*	3.5*	13.0*	18.6*	25.3*
	Red Kurtosis	7.4*	4.7*	21.8*	2.7*	6.1*
	Red Skew	21.0*	9.4*	29.4*	64.8*	11.3*
	Red Entropy	23.8*	23.8*	23.7*	23.9*	23.8*
	Green	73.3*	37.7*	12.5*	78.6*	23.1*
	Green STD	17.4*	7.1*	18.3*	18.6*	25.3*
	Green Kurtosis	4.5*	3.5*	5.9*	2.7*	6.1*
	Green Skew	61.6*	28.6*	60.8*	64.8*	11.3*
	Green Entropy	24.0*	23.9*	23.8*	23.9*	23.8*
	Blue	105.5*	41.7*	11.0*	78.6*	23.1*
	Blue STD	37.2*	7.3*	10.5*	18.6*	25.3*
	Blue Kurtosis	51.1*	10.5*	21.0*	2.7*	6.1*
	Blue Skew	123.9*	37.3*	29.2*	64.8*	11.3*
	Blue Entropy	23.8*	23.8*	23.7*	23.9*	23.8*
	Contrast	55.9*	36.8*	33.2*	59.5*	27.2*
	Dissimilarity	41.6*	33.3*	31.9*	44.5*	26.9*
	Homogeneity	56.8*	52.0*	54.7*	56.8*	47.4*
	ASM	**142.5***	**152.2***	**119.5***	**141.6***	**141.0***
	Correlation	66.5*	43.1*	42.4*	68.8*	46.3*
**COADREAD** n = 495 slides, 8 sites	Red	57.9*	28.4*	19.5*	104.1*	3.4* (p = 0.001)
	Red STD	34.9*	2.2* (p = 0.031)	45.3*	26.1*	3.5* (p = 0.001)
	Red Kurtosis	13.9*	14.9*	22.6*	18.9*	7.5*
	Red Skew	35.6*	30.5*	19.2*	44.4*	11.2*
	Red Entropy	25.9*	26.0*	26.2*	25.0*	26.1*
	Green	76.0*	8.0*	22.4*	104.1*	3.4* (p = 0.001)
	Green STD	16.9*	0.7 (p = 0.664)	31.1*	26.1*	3.5* (p = 0.001)
	Green Kurtosis	18.3*	4.6*	10.6*	18.9*	7.5*
	Green Skew	34.5*	12.3*	24.7*	44.4*	11.2*
	Green Entropy	24.7*	25.9*	26.1*	25.0*	26.1*
	Blue	232.7*	8.6*	9.2*	104.1*	3.4* (p = 0.001)
	Blue STD	77.4*	0.9 (p = 0.497)	26.7*	26.1*	3.5* (p = 0.001)
	Blue Kurtosis	1.7 (p = 0.098)	1.9 (p = 0.07)	29.7*	18.9*	7.5*
	Blue Skew	125.7*	13.3*	32.5*	44.4*	11.2*
	Blue Entropy	25.1*	25.9*	25.9*	25.0*	26.1*
	Contrast	13.3*	13.9*	12.7*	16.3*	5.8*
	Dissimilarity	28.2*	27.7*	28.4*	31.1*	12.9*
	Homogeneity	83.4*	83.2*	86.2*	83.3*	76.1*
	ASM	**149.8***	**160.5***	**149.8***	**149.3***	**149.4***
	Correlation	36.3*	22.8*	19.2*	36.0*	8.2*
**HNSC** n = 228 slides, 4 sites	Red	19.2*	12.1*	12.4*	23.6*	1.2 (p = 0.311)
	Red STD	3.1* (p = 0.026)	5.5* (p = 0.001)	13.7*	11.2*	12.6*
	Red Kurtosis	18.1*	14.8*	8.1*	19.5*	3.9* (p = 0.01)
	Red Skew	13.6*	8.9*	5.0* (p = 0.002)	15.0*	3.5* (p = 0.016)

	Red Entropy	1.6 (p = 0.19)	1.6 (p = 0.192)	1.6 (p = 0.189)	1.5 (p = 0.221)	1.5 (p = 0.204)
	Green	31.6*	17.8*	19.6*	23.6*	1.2 (p = 0.311)
	Green STD	9.4*	11.4*	13.7*	11.2*	12.6*
	Green Kurtosis	17.3*	13.7*	5.6* (p = 0.001)	19.5*	3.9* (p = 0.01)
	Green Skew	16.6*	3.3* (p = 0.021)	7.1*	15.0*	3.5* (p = 0.016)
	Green Entropy	1.4 (p = 0.233)	1.5 (p = 0.21)	1.6 (p = 0.196)	1.5 (p = 0.221)	1.5 (p = 0.204)
	Blue	10.7*	21.3*	16.4*	23.6*	1.2 (p = 0.311)
	Blue STD	21.8*	11.9*	37.4*	11.2*	12.6*
	Blue Kurtosis	22.5*	4.8* (p = 0.003)	9.7*	19.5*	3.9* (p = 0.01)
	Blue Skew	12.1*	4.1* (p = 0.007)	8.7*	15.0*	3.5* (p = 0.016)
	Blue Entropy	1.5 (p = 0.218)	1.5 (p = 0.204)	1.6 (p = 0.195)	1.5 (p = 0.221)	1.5 (p = 0.204)
	Contrast	19.0*	13.8*	15.6*	19.1*	3.6* (p = 0.015)
	Dissimilarity	29.4*	21.9*	25.2*	29.2*	10.1*
	Homogeneity	**40.3***	**40.6***	**40.7***	**40.1***	**39.9***
	ASM	12.0*	12.4*	11.9*	12.0*	11.8*
	Correlation	28.1*	20.7*	17.1*	24.4*	6.9*
**LUAD** n = 355 slides, 8 sites	Red	20.1*	21.2*	10.9*	**46.9***	11.7*
	Red STD	14.8*	5.1*	12.5*	10.6*	12.1*
	Red Kurtosis	4.6*	2.1* (p = 0.044)	5.6*	8.9*	11.4*
	Red Skew	15.7*	10.9*	9.6*	32.0*	7.5*
	Red Entropy	24.9*	25.2*	25.1*	24.5*	25.2*
	Green	**51.3***	16.8*	20.1*	46.9*	11.7*
	Green STD	9.8*	4.6*	19.5*	10.6*	12.1*
	Green Kurtosis	10.3*	8.0*	10.3*	8.9*	11.4*
	Green Skew	32.8*	20.4*	**29.6***	32.0*	7.5*
	Green Entropy	23.8*	**25.3***	25.2*	24.5*	25.2*
	Blue	45.6*	17.0*	18.5*	46.9*	11.7*
	Blue STD	14.3*	4.2*	17.8*	10.6*	12.1*
	Blue Kurtosis	4.5*	2.9* (p = 0.007)	7.5*	8.9*	11.4*
	Blue Skew	27.5*	16.9*	12.9*	32.0*	7.5*
	Blue Entropy	24.8*	25.2*	25.2*	24.5*	**25.2***
	Contrast	14.9*	10.7*	12.2*	15.0*	5.8*
	Dissimilarity	14.1*	9.0*	10.4*	14.3*	4.4*
	Homogeneity	11.9*	11.5*	11.6*	12.0*	11.0*
	ASM	9.7*	10.7*	8.5*	9.7*	9.6*
	Correlation	21.5*	15.5*	16.4*	22.8*	13.3*
**LUSC** n = 306 slides, 8 sites	Red	20.1*	21.2*	10.9*	**46.9***	11.7*
	Red STD	14.8*	5.1*	12.5*	10.6*	12.1*
	Red Kurtosis	4.6*	2.1* (p = 0.044)	5.6*	8.9*	11.4*
	Red Skew	15.7*	10.9*	9.6*	32.0*	7.5*
	Red Entropy	24.9*	25.2*	25.1*	24.5*	**25.2***
	Green	51.3*	16.8*	20.1*	**46.9***	11.7*
	Green STD	9.8*	4.6*	19.5*	10.6*	12.1*
	Green Kurtosis	10.3*	8.0*	10.3*	8.9*	11.4*
	Green Skew	32.8*	20.4*	**29.6***	32.0*	7.5*
	Green Entropy	23.8*	**25.3***	25.2*	24.5*	**25.2***
	Blue	**45.6***	17.0*	18.5*	**46.9***	11.7*
	Blue STD	14.3*	4.2*	17.8*	10.6*	12.1*
	Blue Kurtosis	4.5*	2.9* (p = 0.007)	7.5*	8.9*	11.4*

	Blue Skew	27.5*	16.9*	12.9*	32.0*	7.5*
	Blue Entropy	24.8*	25.2*	25.2*	24.5*	25.2*
	Contrast	14.9*	10.7*	12.2*	15.0*	5.8*
	Dissimilarity	14.1*	9.0*	10.4*	14.3*	4.4*
	Homogeneity	11.9*	11.5*	11.6*	12.0*	11.0*
	ASM	9.7*	10.7*	8.5*	9.7*	9.6*
	Correlation	21.5*	15.5*	16.4*	22.8*	13.3*
**KIRC** n = 456 slides, 7 sites	Red	15.5*	21.5*	1.8 (p = 0.096)	25.5*	16.6*
	Red STD	23.6*	15.4*	4.4*	42.1*	16.0*
	Red Kurtosis	19.6*	6.2*	19.4*	13.8*	12.0*
	Red Skew	20.3*	14.7*	22.3*	11.4*	7.0*
	Red Entropy	27.3*	27.8*	27.8*	26.3*	28.0*
	Green	24.0*	11.8*	1.6 (p = 0.142)	25.5*	16.6*
	Green STD	39.1*	17.5*	12.9*	42.1*	16.0*
	Green Kurtosis	11.4*	7.7*	14.7*	13.8*	12.0*
	Green Skew	11.3*	9.3*	11.2*	11.4*	7.0*
	Green Entropy	25.5*	28.1*	27.9*	26.3*	28.0*
	Blue	30.7*	9.7*	3.9*	25.5*	16.6*
	Blue STD	49.8*	16.9*	11.2*	42.1*	16.0*
	Blue Kurtosis	13.6*	8.5*	19.7*	13.8*	12.0*
	Blue Skew	8.4*	6.8*	11.8*	11.4*	7.0*
	Blue Entropy	26.6*	27.9*	27.8*	26.3*	28.0*
	Contrast	36.1*	14.6*	15.9*	39.1*	8.3*
	Dissimilarity	40.8*	20.9*	23.5*	42.6*	11.1*
	Homogeneity	61.7*	59.0*	59.2*	61.8*	57.1*
	ASM	**102.2***	**102.5***	**96.7***	**102.1***	**101.2***
	Correlation	10.7*	13.2*	8.0*	9.6*	7.9*

(*) indicates p-value remained significant even with Benjamini-Hochberg correction for a false discovery rate of 0.05 (calculated per disease type, per stain normalization method). Factor with highest associated F-statistic indicated in bold. Degrees of freedom for each ANOVA test is number of sites listed – 1. Exact p-values listed for tests with p-value > 0.001. Additional p-values and corrected p-values available in source data.

Supplementary Table 3. One-Versus-Rest Area under the Receiver Operating Characteristic Curve (AUROC) for Prediction of Tissue Submitting Site, with 3-Fold Cross Validation. The first p-value (compared to baseline) indicating a two sided paired t-test between the AUROCs generated by the listed method of stain normalization / augmentation, and the values obtained without stain normalization / augmentation. The second p-value (compared to 0.500) indicates a one sided t-test comparing the AUROCs generated by the listed method to stain normalization / augmentation to random chance (AUROC 0.500). The p-values are Benjamini-Hochberg corrected for a false discovery rate of 0.05

Dataset	Slide Adjustment Method	Average AUROC (Range)	p-value (Compared to Baseline)	p-value (Compared to 0.500)
**BRCA** n = 1032 slides, 39 sites	Baseline	0.987 (0.968 - 0.998)	---	1.21×10^{-46} *
	Macenko	0.966 (0.950 - 0.983)	6.36×10^{-12} *	1.70×10^{-47} *
	Reinhard	0.984 (0.962 - 0.992)	1.01×10^{-1}	5.57×10^{-51} *
	Grayscale	0.950 (0.925 - 0.966)	2.79×10^{-20} *	1.21×10^{-46} *
	Grayscale Normalized	0.958 (0.932 - 0.973)	8.19×10^{-15} *	1.45×10^{-44} *
	Light HSV Augmentation	0.973 (0.956 - 0.985)	7.53×10^{-9} *	7.89×10^{-51} *
	Heavy HSV Augmentation	0.965 (0.947 - 0.976)	1.65×10^{-13} *	2.20×10^{-49} *
**COADREAD** n = 579 slides, 23 sites	Baseline	0.986 (0.971 - 0.997)	---	1.87×10^{-49} *
	Macenko	0.975 (0.948 - 0.990)	8.29×10^{-5} *	3.88×10^{-45} *
	Reinhard	0.973 (0.933 - 0.992)	8.00×10^{-4} *	1.86×10^{-39} *
	Grayscale	0.962 (0.917 - 0.983)	1.50×10^{-9} *	1.08×10^{-40} *
	Grayscale Normalized	0.956 (0.890 - 0.982)	5.17×10^{-7} *	7.06×10^{-35} *
	Light HSV Augmentation	0.968 (0.935 - 0.986)	1.12×10^{-7} *	1.60×10^{-42} *
	Heavy HSV Augmentation	0.967 (0.922 - 0.990)	1.09×10^{-6} *	4.56×10^{-40} *
**HNSC** n = 431 slides, 26 sites	Baseline	0.965 (0.899 - 0.992)	---	4.01×10^{-33} *
	Macenko	0.935 (0.905 - 0.965)	3.07×10^{-5} *	6.55×10^{-41} *
	Reinhard	0.915 (0.867 - 0.962)	8.87×10^{-9} *	1.16×10^{-35} *
	Grayscale	0.853 (0.786 - 0.920)	9.04×10^{-16} *	4.48×10^{-27} *
	Grayscale Normalized	0.870 (0.818 - 0.906)	9.93×10^{-17} *	1.93×10^{-32} *
	Light HSV Augmentation	0.920 (0.836 - 0.974)	2.48×10^{-5} *	6.28×10^{-29} *
	Heavy HSV Augmentation	0.901 (0.807 - 0.961)	2.71×10^{-8} *	2.41×10^{-28} *
**LUAD** n = 458 slides, 33 sites	Baseline	0.967 (0.917 - 0.993)	---	4.68×10^{-34} *
	Macenko	0.955 (0.916 - 0.984)	1.01×10^{-1}	5.78×10^{-34} *
	Reinhard	0.957 (0.922 - 0.980)	1.49×10^{-1}	1.65×10^{-34} *
	Grayscale	0.895 (0.832 - 0.939)	1.83×10^{-11} *	4.70×10^{-28} *
	Grayscale Normalized	0.952 (0.901 - 0.996)	1.01×10^{-1}	4.70×10^{-28} *
	Light HSV Augmentation	0.939 (0.915 - 0.968)	8.41×10^{-5} *	1.73×10^{-34} *
	Heavy HSV Augmentation	0.925 (0.891 - 0.965)	8.22×10^{-7} *	3.43×10^{-31} *
**LUSC** n = 463 slides, 35 sites	Baseline	0.964 (0.905 - 0.994)	---	1.31×10^{-33} *
	Macenko	0.950 (0.913 - 0.976)	5.11×10^{-2}	3.36×10^{-38} *
	Reinhard	0.957 (0.925 - 0.978)	3.22×10^{-1}	4.56×10^{-40} *
	Grayscale	0.925 (0.890 - 0.963)	1.46×10^{-7} *	1.74×10^{-38} *
	Grayscale Normalized	0.931 (0.906 - 0.957)	2.13×10^{-6} *	2.27×10^{-41} *
	Light HSV Augmentation	0.934 (0.876 - 0.975)	2.80×10^{-4} *	4.84×10^{-33} *
	Heavy HSV Augmentation	0.935 (0.901 - 0.965)	3.10×10^{-5} *	2.23×10^{-39} *

**KIRC** n = 505 slides, 17 sites	Baseline	0.998 (0.995 - 1.000)	---	7.85×10^{-70} *
	Macenko	0.992 (0.981 - 0.999)	1.95×10^{-10} *	1.04×10^{-56} *
	Reinhard	0.992 (0.982 - 0.997)	8.44×10^{-10} *	5.16×10^{-56} *
	Grayscale	0.925 (0.854 - 0.967)	4.62×10^{-16} *	1.05×10^{-31} *
	Grayscale Normalized	0.998 (0.993 - 1.000)	8.85×10^{-1}	5.52×10^{-64} *
	Light HSV Augmentation	0.973 (0.954 - 0.987)	5.48×10^{-19} *	1.20×10^{-46} *
	Heavy HSV Augmentation	0.960 (0.928 - 0.988)	1.22×10^{-17} *	1.08×10^{-40} *

P-values listed are Benjamini-Hochberg corrected for a false discovery rate of 0.05, with (*) indicating p-values maintain significance with correction.

Supplementary Table 4. Prediction of Tissue Submitting Site Using Demographic, Clinical, and Genomic Features.

Dataset	# Sites	Average AUROC, 3-Fold Cross Validation
BRCA	15	0.642
COADREAD	15	0.781
HNSC	19	0.613
KIRC	12	0.600
LUAD	26	0.591
LUSC	27	0.511

Supplementary Table 5. Accuracy in Predicting a Synthetic Feature, as a Function of Biologic / Stain related Confounders.

Normalization	ER Negative Slides	Stain Altered Slides				
		**0**	**5**	**11**	**17**	**23**
Baseline	0	0.49	0.532	0.659	0.822	1.000
	5	0.488	0.521	0.714	0.854	1.000
	11	0.597	0.625	0.715	0.919	1.000
	17	0.638	0.644	0.715	0.863	1.000
	23	0.664	0.684	0.774	0.871	1.000
Reinhard	0	0.454	0.578	0.673	0.810	1.000
	5	0.501	0.602	0.753	0.893	0.992
	11	0.547	0.599	0.779	0.903	0.987
	17	0.636	0.634	0.755	0.842	0.985
	23	0.705	0.720	0.786	0.839	0.997
Macenko	0	0.489	0.558	0.748	0.881	1.000
	5	0.473	0.553	0.769	0.910	1.000
	11	0.416	0.573	0.707	0.863	1.000
	17	0.617	0.636	0.706	0.844	1.000
	23	0.746	0.784	0.837	0.893	1.000
Grayscale	0	0.406	0.430	0.553	0.711	0.946
	5	0.443	0.449	0.607	0.764	0.959
	11	0.443	0.506	0.601	0.764	0.968
	17	0.610	0.645	0.714	0.740	0.934
	23	0.624	0.645	0.683	0.725	0.934
Grayscale Normalized	0	0.412	0.455	0.560	0.755	0.988
	5	0.547	0.572	0.685	0.863	0.988
	11	0.585	0.581	0.732	0.858	0.971
	17	0.609	0.637	0.726	0.819	0.953
	23	0.725	0.747	0.783	0.788	0.976
Augment	0	0.395	0.481	0.639	0.775	0.997
	5	0.391	0.459	0.643	0.837	0.994
	11	0.582	0.649	0.743	0.868	0.997
	17	0.578	0.617	0.702	0.786	0.965
	23	0.552	0.604	0.685	0.789	0.997
Augment Heavy	0	0.414	0.511	0.661	0.801	1.000
	5	0.445	0.471	0.674	0.808	1.000
	11	0.511	0.601	0.747	0.865	1.000
	17	0.606	0.606	0.739	0.826	0.985
	23	0.646	0.660	0.712	0.796	0.997

Supplementary Table 6. Distribution of Patients Across Validation Folds with Standard and Preserved Site Cross Validation.

Dataset	Feature	# Slides	# Slides Excluded	Subgroup	Standard Cross Validation			Preserved Site Cross Validation		
					CV1	CV2	CV3	CV1	CV2	CV3
BRCA	Age	1062	9	<39	25	25	25	25	25	25
				40-59	163	162	162	163	163	162
				60+	167	166	166	166	166	167
	Stage	1044	8	Stage I	59	59	58	58	59	59
				Stage II	203	203	202	203	202	203
				Stage III	81	80	80	80	80	81
				Stage IV	7	6	6	7	6	6
	ER Status	1011	8	Positive	261	261	261	261	261	261
				Negative	76	76	76	76	76	76
	PR Status	1008	8	Positive	225	225	224	225	225	224
				Negative	112	111	111	111	112	111
	HER2 Status	856	2	Positive	50	50	50	50	50	50
				Negative	236	235	235	236	235	235
	PAM50 Subtype	950	10	Luminal	225	224	224	224	224	225
				Basal	56	56	55	56	55	56
				HER2	26	25	25	27	24	25
				Normal	12	11	11	11	12	11
	Histologic Subtype	948	8	IDC	253	252	252	253	252	252
				ILC	64	64	63	63	64	64
	Ancestry	913	8	African	54	54	54	54	54	54
				European	251	250	250	250	251	250
	PFS	489	2	≤3 years	29	29	28	29	29	28
				>3 years	135	134	134	134	135	134
	TP53	1048	8	Driver	113	113	112	113	112	113
				No Driver	237	237	236	237	236	237
	MAP3K1	1048	8	Driver	23	22	22	22	23	22
				No Driver	327	327	327	327	327	327
	BRCA1	931	8	Mutation	11	10	10	11	10	10
				No Mutation	300	300	300	300	300	300
	Immune Subtype	1007	8	C1	118	117	117	117	117	118
				C2	128	127	127	127	127	128
				C3	62	62	61	62	61	62
				C4	30	29	29	29	30	29
COADREAD	Age	562	21	40-59	50	50	50	36	57	57
				60+	138	137	137	192	110	110
	Stage	594	26	Stage I	36	36	35	55	26	26
				Stage II	75	75	74	98	63	63
				Stage III	59	59	58	61	57	58
				Stage IV	29	29	29	38	24	25
	Histologic Subtype	604	26	Mucinous	25	24	24	29	22	22
				Not Mucinous	177	177	177	220	155	156
	Cancer Subsite	604	26	Colon	149	149	148	137	172	137
				Rectal	53	53	52	41	77	40

	**Ancestry**	504	21	African	20	20	20	20	20	20
				European	148	148	148	132	180	132
	**BMI**	298	1	<25	31	31	31	31	31	31
				25-34.9	56	55	55	56	55	55
				35+	13	13	13	13	13	13
	**MSI Status**	273	7	MSS	64	64	64	164	17	11
				MSI	27	27	27	66	7	8
	**PFS**	248	11	≤3 years	46	45	45	50	43	43
				>3 years	38	37	37	40	36	36
	**RNF43**	577	21	Driver	12	11	11	14	6	14
				No Driver	181	181	181	160	223	160
	**BRAF**	577	21	Driver	18	18	17	16	19	18
				No Driver	175	175	174	213	156	155
	**Immune Subtype**	576	22	C1	148	148	148	135	173	136
				C2	34	34	33	28	45	28
				C3	6	6	6	3	12	3
				C4	5	4	4	4	4	5
**HNSC**	**Age**	435	7	40-59	61	61	61	62	59	62
				60+	84	84	84	80	91	81
	**Stage**	400	7	Stage I	87	87	87	87	87	87
				Stage II	23	22	22	19	30	18
				Stage III	24	24	24	24	24	24
	**Gender**	450	7	Male	110	109	109	109	110	109
				Female	41	41	40	42	40	40
	**Grade**	428	7	G1	19	19	18	17	21	18
				G2	89	88	88	89	88	88
				G3	36	36	35	34	40	33
	**HPV Status**	437	2	Negative	129	129	129	130	129	128
				Positive	17	17	16	15	18	17
	**Ancestry**	402	2	African	15	14	14	14	14	15
				European	120	120	119	119	120	120
	**PFS**	274	2	≤3 years	52	52	52	52	52	52
				>3 years	40	39	39	36	45	37
	**TP53**	446	3	Driver	107	107	106	108	106	106
				No Driver	42	42	42	42	42	42
	**RHOA**	446	3	Driver	2	2	1	2	2	1
				No Driver	147	147	147	147	147	147
	**Immune Subtype**	433	2	C1	38	38	37	36	38	39
				C2	107	107	106	106	107	107
**KIRC**	**Age**	513	0	<39	6	5	5	7	5	4
				40-59	74	73	73	68	83	69
				60+	93	92	92	94	92	91
	**Stage**	510	0	Stage I	86	85	85	96	78	82
				Stage II	19	19	19	22	19	16
				Stage III	40	40	39	41	35	43
				Stage IV	26	26	26	19	30	29
	**Gender**	513	0	Male	111	110	110	115	108	108
				Female	61	61	60	60	61	61
	**Grade**	505	0	G1	5	4	4	3	6	4
				G2	72	72	72	80	68	68
				G3	68	67	67	65	68	69

				G4	25	25	24	21	26	27
	Ancestry	426	0	African	16	16	15	15	16	16
				European	127	126	126	127	125	127
	PFS	346	0	≤3 years	39	39	38	37	44	35
				>3 years	77	77	76	81	74	75
	Immune Subtype	449	0	C3	141	141	140	141	141	140
				C4	9	9	9	9	9	9
LUAD	Age	446	10	40-59	42	42	41	42	41	42
				60+	111	110	110	110	111	110
	Stage	478	10	Stage I	89	89	88	88	89	89
				Stage II	39	39	39	39	39	39
				Stage III	23	23	23	23	23	23
				Stage IV	9	9	8	9	8	9
	Gender	478	10	Male	74	74	74	74	74	74
				Female	86	85	85	85	86	85
	ALK Fusion	231	0	Absent	67	67	66	67	66	67
				Present	11	10	10	10	10	11
	Ancestry	434	6	African	16	16	16	16	16	16
				European	129	129	128	128	129	129
	PFS	258	3	≤3 years	57	56	56	56	56	57
				>3 years	30	30	29	29	30	30
	TP53	471	9	Driver	82	82	81	82	81	82
				No Driver	76	75	75	75	76	75
	STK11	471	9	Driver	22	22	22	22	22	22
				No Driver	135	135	135	135	135	135
	Immune Subtype	393	7	C1	26	26	25	26	25	26
				C2	45	45	45	45	45	45
				C3	54	54	54	54	54	54
				C4	7	6	6	7	6	6
LUSC	Age	468	4	40-59	29	28	28	28	28	29
				60+	128	128	127	128	128	127
	Stage	478	4	Stage I	80	79	79	80	79	79
				Stage II	51	51	51	51	51	51
				Stage III	27	27	27	27	27	27
				Stage IV	2	2	2	2	2	2
	Gender	478	4	Male	119	119	119	119	119	119
				Female	41	40	40	40	41	40
	ALK Fusion	273	3	Absent	89	88	88	89	88	88
				Present	3	3	2	5	2	1
	Ancestry	426	4	African	10	10	9	10	9	10
				European	133	132	132	132	133	132
	PFS	231	4	≤3 years	37	37	37	37	37	37
				>3 years	40	40	40	40	40	40
	PIK3R1	462	4	Driver	2	2	1	2	2	1
				No Driver	153	152	152	153	152	152
	Immune Subtype	435	3	C1	88	87	87	88	87	87
				C2	58	58	57	58	58	57

Supplementary Table 7. One-Versus-Rest Area under the Receiver Operating Characteristic Curve (AUROC) for Prediction of Demographic, Clinical, and Genetic Features.

Dataset	Feature	Slide Adjustment Method	Standard Cross Validation AUROC (Range)	Standard Cross Validation p-value, Compared to 0.500	Preserved Site Cross Validation AUROC (Range)	Preserved Site Cross Validation p-value, Compared to 0.500	p-value, Between Group
BRCA	Stage	Baseline	0.595 (0.541 - 0.663)	8.13×10^{-14}	0.557 (0.499 - 0.636)	4.53×10^{-9}	4.74×10^{-4}
		Macenko	0.598 (0.545 - 0.651)	2.75×10^{-16}	0.551 (0.491 - 0.610)	1.93×10^{-9}	1.79×10^{-6}
		Reinhard	0.602 (0.535 - 0.670)	1.67×10^{-17}	0.571 (0.464 - 0.640)	3.62×10^{-9}	3.17×10^{-3}
		Grayscale	0.600 (0.565 - 0.637)	5.73×10^{-20}	0.559 (0.499 - 0.603)	3.87×10^{-12}	1.16×10^{-7}
		Normalized	0.627 (0.575 - 0.701)	8.26×10^{-18}	0.599 (0.551 - 0.651)	4.41×10^{-18}	9.52×10^{-4}
		Light	0.592 (0.542 - 0.641)	2.68×10^{-18}	0.581 (0.522 - 0.629)	7.12×10^{-15}	9.40×10^{-2}
		Heavy	0.603 (0.549 - 0.664)	1.19×10^{-18}	0.590 (0.538 - 0.645)	6.64×10^{-16}	5.44×10^{-2}
	Age	Baseline	0.602 (0.488 - 0.672)	1.38×10^{-12}	0.586 (0.534 - 0.636)	9.36×10^{-15}	1.05×10^{-1}
		Macenko	0.633 (0.545 - 0.701)	5.76×10^{-17}	0.591 (0.547 - 0.642)	7.16×10^{-16}	2.82×10^{-5}
		Reinhard	0.605 (0.534 - 0.677)	9.04×10^{-15}	0.563 (0.512 - 0.645)	3.62×10^{-9}	1.88×10^{-4}
		Grayscale	0.581 (0.497 - 0.633)	7.16×10^{-13}	0.574 (0.502 - 0.636)	4.11×10^{-12}	3.27×10^{-1}
		Normalized	0.619 (0.548 - 0.698)	1.39×10^{-17}	0.570 (0.501 - 0.615)	1.17×10^{-10}	4.11×10^{-6}
		Light	0.617 (0.511 - 0.682)	4.10×10^{-15}	0.613 (0.562 - 0.673)	1.83×10^{-16}	4.34×10^{-1}
		Heavy	0.614 (0.531 - 0.683)	4.94×10^{-16}	0.597 (0.535 - 0.659)	7.21×10^{-14}	6.77×10^{-2}
	ER status	Baseline	0.857 (0.795 - 0.905)	3.93×10^{-29}	0.840 (0.770 - 0.893)	1.05×10^{-26}	6.29×10^{-2}
		Macenko	0.889 (0.814 - 0.936)	2.13×10^{-30}	0.856 (0.806 - 0.905)	1.05×10^{-29}	1.88×10^{-4}
		Reinhard	0.885 (0.837 - 0.925)	2.99×10^{-33}	0.865 (0.814 - 0.915)	4.54×10^{-30}	5.47×10^{-3}
		Grayscale	0.852 (0.781 - 0.901)	1.06×10^{-30}	0.842 (0.741 - 0.916)	1.30×10^{-26}	1.78×10^{-1}
		Normalized	0.894 (0.844 - 0.941)	2.99×10^{-33}	0.870 (0.793 - 0.904)	2.23×10^{-30}	6.98×10^{-4}
		Light	0.866 (0.795 - 0.919)	4.83×10^{-29}	0.847 (0.752 - 0.909)	1.93×10^{-25}	6.38×10^{-2}
		Heavy	0.869 (0.793 - 0.918)	2.13×10^{-30}	0.853 (0.762 - 0.918)	1.67×10^{-26}	7.73×10^{-2}
	PR Status	Baseline	0.746 (0.663 - 0.821)	6.81×10^{-22}	0.722 (0.568 - 0.829)	5.88×10^{-15}	1.14×10^{-1}
		Macenko	0.779 (0.701 - 0.836)	5.14×10^{-24}	0.764 (0.699 - 0.825)	3.69×10^{-24}	1.23×10^{-1}
		Reinhard	0.770 (0.703 - 0.834)	8.39×10^{-25}	0.794 (0.758 - 0.867)	2.67×10^{-27}	9.95×10^{-1}
		Grayscale	0.763 (0.691 - 0.814)	1.66×10^{-25}	0.715 (0.620 - 0.780)	6.49×10^{-22}	9.30×10^{-6}
		Normalized	0.796 (0.740 - 0.836)	2.71×10^{-28}	0.773 (0.714 - 0.836)	6.70×10^{-25}	1.08×10^{-2}
		Light	0.751 (0.676 - 0.813)	7.66×10^{-24}	0.754 (0.689 - 0.843)	5.09×10^{-23}	6.74×10^{-1}
		Heavy	0.773 (0.698 - 0.831)	2.62×10^{-24}	0.770 (0.710 - 0.862)	7.42×10^{-24}	4.62×10^{-1}
	HER2 Status	Baseline	0.616 (0.505 - 0.709)	5.46×10^{-12}	0.603 (0.478 - 0.720)	3.08×10^{-9}	2.96×10^{-1}
		Macenko	0.652 (0.573 - 0.724)	2.74×10^{-18}	0.651 (0.517 - 0.734)	3.00×10^{-15}	5.63×10^{-1}
		Reinhard	0.626 (0.516 - 0.689)	3.32×10^{-15}	0.650 (0.511 - 0.789)	3.92×10^{-11}	9.83×10^{-1}
		Grayscale	0.600 (0.481 - 0.705)	1.87×10^{-10}	0.632 (0.522 - 0.733)	1.04×10^{-12}	9.95×10^{-1}
		Normalized	0.656 (0.527 - 0.765)	1.50×10^{-13}	0.684 (0.617 - 0.761)	1.63×10^{-19}	9.95×10^{-1}
		Light	0.589 (0.504 - 0.650)	2.21×10^{-11}	0.576 (0.457 - 0.674)	2.60×10^{-8}	2.36×10^{-1}
		Heavy	0.601 (0.484 - 0.675)	8.75×10^{-12}	0.594 (0.513 - 0.667)	7.23×10^{-12}	3.62×10^{-1}
	Histologic Subtype	Baseline	0.900 (0.808 - 0.966)	2.22×10^{-26}	0.835 (0.688 - 0.945)	1.06×10^{-17}	1.37×10^{-3}
		Macenko	0.929 (0.864 - 0.968)	3.85×10^{-33}	0.923 (0.826 - 0.968)	2.30×10^{-28}	3.27×10^{-1}
		Reinhard	0.920 (0.850 - 0.970)	7.93×10^{-31}	0.918 (0.862 - 0.965)	7.59×10^{-30}	4.63×10^{-1}
		Grayscale	0.900 (0.842 - 0.953)	3.08×10^{-31}	0.901 (0.816 - 0.969)	1.09×10^{-26}	6.13×10^{-1}
		Normalized	0.928 (0.889 - 0.964)	2.41×10^{-35}	0.918 (0.862 - 0.960)	2.23×10^{-30}	1.22×10^{-1}
		Light	0.911 (0.864 - 0.964)	1.98×10^{-32}	0.894 (0.814 - 0.946)	8.79×10^{-26}	9.40×10^{-2}
		Heavy	0.912 (0.871 - 0.952)	2.16×10^{-33}	0.908 (0.840 - 0.959)	1.14×10^{-26}	4.36×10^{-1}
		Baseline	0.740 (0.667 - 0.818)	2.59×10^{-22}	0.733 (0.653 - 0.830)	4.62×10^{-20}	3.62×10^{-1}

PAM50 Subtype	Macenko	0.810 (0.741 - 0.863)	9.61×10^{-27}	0.794 (0.717 - 0.869)	2.82×10^{-22}	1.23×10^{-1}
	Reinhard	0.804 (0.747 - 0.850)	5.78×10^{-28}	0.782 (0.696 - 0.867)	1.66×10^{-21}	5.05×10^{-2}
	Grayscale	0.754 (0.656 - 0.807)	7.66×10^{-24}	0.748 (0.670 - 0.837)	1.02×10^{-20}	3.70×10^{-1}
	Normalized	0.817 (0.761 - 0.867)	1.49×10^{-28}	0.788 (0.717 - 0.883)	3.12×10^{-24}	3.88×10^{-3}
	Light	0.771 (0.728 - 0.831)	6.29×10^{-27}	0.770 (0.638 - 0.861)	1.37×10^{-19}	5.64×10^{-1}
	Heavy	0.790 (0.742 - 0.837)	2.71×10^{-28}	0.782 (0.672 - 0.877)	2.76×10^{-21}	3.10×10^{-1}
Immune Subtype	Baseline	0.645 (0.599 - 0.689)	3.34×10^{-22}	0.585 (0.509 - 0.631)	1.00×10^{-14}	1.89×10^{-10}
	Macenko	0.679 (0.631 - 0.713)	4.94×10^{-25}	0.653 (0.589 - 0.734)	8.66×10^{-20}	1.61×10^{-3}
	Reinhard	0.689 (0.636 - 0.730)	2.48×10^{-26}	0.644 (0.591 - 0.708)	4.54×10^{-19}	6.84×10^{-7}
	Grayscale	0.681 (0.643 - 0.719)	1.66×10^{-26}	0.652 (0.613 - 0.694)	3.33×10^{-24}	9.03×10^{-6}
	Normalized	0.694 (0.642 - 0.742)	1.47×10^{-26}	0.670 (0.621 - 0.708)	2.45×10^{-23}	6.38×10^{-4}
	Light	0.671 (0.611 - 0.705)	2.38×10^{-23}	0.629 (0.586 - 0.679)	1.55×10^{-19}	1.68×10^{-6}
	Heavy	0.684 (0.638 - 0.735)	5.14×10^{-24}	0.649 (0.596 - 0.698)	1.11×10^{-21}	1.45×10^{-5}
PFS	Baseline	0.586 (0.436 - 0.688)	1.19×10^{-7}	0.538 (0.383 - 0.694)	1.14×10^{-2}	1.90×10^{-2}
	Macenko	0.546 (0.394 - 0.708)	3.42×10^{-3}	0.586 (0.436 - 0.709)	1.55×10^{-7}	9.95×10^{-1}
	Reinhard	0.599 (0.415 - 0.719)	9.29×10^{-8}	0.620 (0.445 - 0.738)	4.81×10^{-9}	9.12×10^{-1}
	Grayscale	0.502 (0.366 - 0.648)	4.29×10^{-1}	0.516 (0.372 - 0.638)	1.07×10^{-1}	8.51×10^{-1}
	Normalized	0.576 (0.375 - 0.713)	4.80×10^{-5}	0.555 (0.472 - 0.681)	3.91×10^{-5}	2.15×10^{-1}
	Light	0.520 (0.354 - 0.688)	9.87×10^{-2}	0.537 (0.376 - 0.657)	5.12×10^{-3}	8.72×10^{-1}
	Heavy	0.531 (0.382 - 0.697)	3.04×10^{-2}	0.569 (0.438 - 0.658)	3.27×10^{-6}	9.95×10^{-1}
BRCA1 Mutation	Baseline	0.723 (0.395 - 0.890)	5.35×10^{-12}	0.629 (0.481 - 0.852)	1.69×10^{-7}	1.54×10^{-3}
	Macenko	0.666 (0.258 - 0.850)	7.64×10^{-7}	0.623 (0.428 - 0.787)	4.28×10^{-7}	1.45×10^{-1}
	Reinhard	0.694 (0.295 - 0.933)	5.14×10^{-9}	0.570 (0.417 - 0.780)	4.13×10^{-4}	2.56×10^{-4}
	Grayscale	0.609 (0.424 - 0.812)	1.62×10^{-7}	0.526 (0.370 - 0.714)	7.71×10^{-2}	1.30×10^{-3}
	Normalized	0.614 (0.365 - 0.809)	6.57×10^{-6}	0.586 (0.319 - 0.792)	4.17×10^{-4}	2.60×10^{-1}
	Light	0.661 (0.232 - 0.890)	1.94×10^{-6}	0.537 (0.395 - 0.670)	9.62×10^{-3}	4.35×10^{-4}
	Heavy	0.613 (0.300 - 0.847)	2.04×10^{-5}	0.525 (0.364 - 0.717)	1.11×10^{-1}	6.89×10^{-3}
TP53	Baseline	0.769 (0.676 - 0.822)	6.53×10^{-26}	0.752 (0.695 - 0.853)	3.74×10^{-21}	1.01×10^{-1}
	Macenko	0.805 (0.732 - 0.859)	1.78×10^{-28}	0.788 (0.697 - 0.870)	1.37×10^{-21}	1.22×10^{-1}
	Reinhard	0.794 (0.718 - 0.862)	4.53×10^{-25}	0.790 (0.705 - 0.857)	4.60×10^{-24}	4.16×10^{-1}
	Grayscale	0.794 (0.723 - 0.848)	3.80×10^{-26}	0.781 (0.726 - 0.850)	4.60×10^{-24}	1.41×10^{-1}
	Normalized	0.804 (0.736 - 0.880)	4.75×10^{-26}	0.804 (0.739 - 0.872)	1.67×10^{-26}	5.84×10^{-1}
	Light	0.797 (0.724 - 0.857)	2.16×10^{-26}	0.777 (0.729 - 0.853)	3.33×10^{-24}	4.68×10^{-2}
	Heavy	0.809 (0.747 - 0.874)	4.75×10^{-26}	0.784 (0.736 - 0.851)	1.49×10^{-25}	1.26×10^{-2}
MAP3K1	Baseline	0.654 (0.503 - 0.788)	1.97×10^{-10}	0.585 (0.428 - 0.725)	8.56×10^{-7}	2.48×10^{-3}
	Macenko	0.679 (0.539 - 0.797)	2.82×10^{-12}	0.681 (0.524 - 0.809)	2.39×10^{-13}	6.13×10^{-1}
	Reinhard	0.684 (0.531 - 0.814)	6.05×10^{-12}	0.660 (0.515 - 0.793)	1.07×10^{-12}	1.80×10^{-1}
	Grayscale	0.693 (0.607 - 0.834)	1.81×10^{-15}	0.627 (0.553 - 0.690)	4.05×10^{-15}	4.90×10^{-5}
	Normalized	0.773 (0.659 - 0.876)	2.44×10^{-20}	0.669 (0.489 - 0.837)	9.62×10^{-10}	2.49×10^{-5}
	Light	0.669 (0.579 - 0.761)	3.32×10^{-15}	0.634 (0.542 - 0.784)	7.12×10^{-13}	2.42×10^{-2}
	Heavy	0.726 (0.653 - 0.843)	2.21×10^{-18}	0.642 (0.558 - 0.769)	2.39×10^{-13}	1.92×10^{-6}
Ancestry	Baseline	0.798 (0.719 - 0.893)	6.80×10^{-25}	0.507 (0.306 - 0.713)	3.91×10^{-1}	4.88×10^{-15}
	Macenko	0.627 (0.523 - 0.777)	7.89×10^{-8}	0.558 (0.435 - 0.661)	1.66×10^{-4}	4.84×10^{-3}
	Reinhard	0.792 (0.688 - 0.837)	1.49×10^{-25}	0.638 (0.536 - 0.759)	1.93×10^{-12}	4.06×10^{-15}
	Grayscale	0.738 (0.619 - 0.814)	3.92×10^{-20}	0.581 (0.478 - 0.672)	4.96×10^{-9}	4.14×10^{-15}
	Normalized	0.750 (0.677 - 0.806)	9.77×10^{-25}	0.624 (0.547 - 0.758)	7.12×10^{-13}	3.41×10^{-14}
	Light	0.778 (0.699 - 0.839)	5.11×10^{-25}	0.549 (0.406 - 0.682)	7.59×10^{-4}	3.61×10^{-19}
	Heavy	0.778 (0.662 - 0.852)	6.82×10^{-24}	0.561 (0.456 - 0.696)	2.10×10^{-6}	3.22×10^{-21}
Age	Baseline	0.605 (0.528 - 0.726)	1.71×10^{-10}	0.479 (0.375 - 0.614)	9.82×10^{-1}	1.75×10^{-9}

COAD READ		Macenko	0.571 (0.428 - 0.681)	1.62×10^{-6}	0.483 (0.390 - 0.606)	9.82×10^{-1}	2.34×10^{-6}
		Reinhard	0.621 (0.531 - 0.778)	4.04×10^{-11}	0.475 (0.339 - 0.610)	9.82×10^{-1}	9.47×10^{-10}
		Grayscale	0.587 (0.500 - 0.755)	4.38×10^{-8}	0.527 (0.390 - 0.685)	4.07×10^{-2}	2.13×10^{-3}
		Normalized	0.597 (0.504 - 0.752)	1.50×10^{-9}	0.467 (0.388 - 0.533)	1.00×10^{-0}	2.17×10^{-11}
		Light	0.567 (0.459 - 0.669)	5.70×10^{-7}	0.487 (0.334 - 0.598)	9.54×10^{-1}	8.35×10^{-6}
		Heavy	0.579 (0.465 - 0.725)	1.07×10^{-7}	0.486 (0.359 - 0.619)	9.61×10^{-1}	9.35×10^{-7}
	Stage	Baseline	0.586 (0.517 - 0.657)	2.45×10^{-12}	0.565 (0.494 - 0.688)	8.36×10^{-8}	5.27×10^{-2}
		Macenko	0.600 (0.524 - 0.684)	7.57×10^{-12}	0.578 (0.475 - 0.670)	9.65×10^{-9}	6.44×10^{-2}
		Reinhard	0.619 (0.555 - 0.688)	1.54×10^{-15}	0.576 (0.466 - 0.681)	5.06×10^{-7}	2.61×10^{-3}
		Grayscale	0.563 (0.528 - 0.631)	1.06×10^{-12}	0.552 (0.474 - 0.608)	5.45×10^{-9}	1.17×10^{-1}
		Normalized	0.600 (0.550 - 0.660)	7.75×10^{-18}	0.587 (0.522 - 0.643)	4.71×10^{-13}	7.75×10^{-2}
		Light	0.586 (0.539 - 0.633)	2.37×10^{-15}	0.574 (0.492 - 0.650)	2.81×10^{-10}	1.18×10^{-1}
		Heavy	0.588 (0.538 - 0.649)	1.01×10^{-13}	0.566 (0.496 - 0.661)	2.33×10^{-8}	3.81×10^{-2}
	Histologic Subtype	Baseline	0.788 (0.703 - 0.844)	2.97×10^{-24}	0.712 (0.579 - 0.799)	6.15×10^{-16}	2.14×10^{-6}
		Macenko	0.839 (0.719 - 0.898)	2.68×10^{-23}	0.774 (0.686 - 0.865)	4.29×10^{-20}	2.11×10^{-5}
		Reinhard	0.883 (0.794 - 0.958)	7.64×10^{-26}	0.768 (0.612 - 0.883)	1.10×10^{-16}	4.65×10^{-9}
		Grayscale	0.844 (0.755 - 0.948)	2.68×10^{-23}	0.757 (0.667 - 0.842)	4.33×10^{-18}	5.58×10^{-7}
		Normalized	0.854 (0.775 - 0.913)	9.77×10^{-26}	0.829 (0.761 - 0.901)	2.72×10^{-24}	1.95×10^{-2}
		Light	0.827 (0.722 - 0.919)	1.95×10^{-22}	0.767 (0.660 - 0.891)	9.80×10^{-18}	3.22×10^{-4}
		Heavy	0.863 (0.787 - 0.951)	7.75×10^{-25}	0.777 (0.682 - 0.869)	1.17×10^{-19}	1.12×10^{-7}
	Cancer Subsite	Baseline	0.548 (0.387 - 0.682)	6.56×10^{-4}	0.547 (0.406 - 0.660)	1.01×10^{-3}	5.29×10^{-1}
		Macenko	0.582 (0.441 - 0.681)	1.60×10^{-7}	0.573 (0.459 - 0.703)	7.48×10^{-6}	3.77×10^{-1}
		Reinhard	0.601 (0.482 - 0.711)	2.81×10^{-10}	0.593 (0.486 - 0.696)	1.14×10^{-10}	3.51×10^{-1}
		Grayscale	0.616 (0.526 - 0.722)	3.42×10^{-11}	0.595 (0.490 - 0.741)	6.69×10^{-9}	1.26×10^{-1}
		Normalized	0.654 (0.578 - 0.750)	2.69×10^{-17}	0.584 (0.411 - 0.749)	7.85×10^{-6}	1.67×10^{-4}
		Light	0.587 (0.480 - 0.733)	4.56×10^{-8}	0.596 (0.480 - 0.713)	1.07×10^{-7}	7.19×10^{-1}
		Heavy	0.634 (0.536 - 0.731)	2.98×10^{-13}	0.595 (0.494 - 0.698)	2.85×10^{-9}	9.98×10^{-3}
	Ancestry	Baseline	0.883 (0.755 - 0.976)	8.42×10^{-23}	0.795 (0.564 - 0.928)	1.28×10^{-15}	7.23×10^{-5}
		Macenko	0.883 (0.753 - 0.937)	7.68×10^{-26}	0.573 (0.108 - 0.892)	1.21×10^{-1}	2.79×10^{-6}
		Reinhard	0.895 (0.803 - 0.959)	4.01×10^{-25}	0.525 (0.003 - 0.918)	4.24×10^{-1}	9.52×10^{-6}
		Grayscale	0.890 (0.790 - 0.967)	1.39×10^{-24}	0.802 (0.631 - 0.941)	4.41×10^{-16}	2.23×10^{-5}
		Normalized	0.859 (0.734 - 0.981)	1.38×10^{-19}	0.495 (0.117 - 0.805)	6.22×10^{-1}	3.99×10^{-8}
		Light	0.884 (0.803 - 0.955)	4.34×10^{-25}	0.799 (0.615 - 0.942)	2.51×10^{-15}	7.84×10^{-5}
		Heavy	0.891 (0.784 - 0.961)	4.01×10^{-25}	0.827 (0.617 - 0.977)	3.99×10^{-15}	4.18×10^{-3}
	BMI	Baseline	0.551 (0.462 - 0.691)	6.06×10^{-5}	0.475 (0.360 - 0.577)	1.00×10^{-0}	1.41×10^{-5}
		Macenko	0.573 (0.469 - 0.679)	4.19×10^{-8}	0.560 (0.462 - 0.678)	3.45×10^{-5}	2.84×10^{-1}
		Reinhard	0.573 (0.491 - 0.672)	4.90×10^{-8}	0.527 (0.410 - 0.641)	3.46×10^{-2}	7.61×10^{-3}
		Grayscale	0.549 (0.425 - 0.672)	9.50×10^{-5}	0.496 (0.409 - 0.620)	7.18×10^{-1}	2.13×10^{-3}
		Normalized	0.576 (0.507 - 0.689)	1.80×10^{-9}	0.544 (0.434 - 0.635)	3.62×10^{-4}	2.01×10^{-2}
		Light	0.563 (0.452 - 0.680)	2.01×10^{-6}	0.522 (0.421 - 0.618)	4.48×10^{-2}	1.09×10^{-2}
		Heavy	0.551 (0.447 - 0.670)	7.59×10^{-5}	0.539 (0.412 - 0.653)	6.48×10^{-3}	3.16×10^{-1}
	PFS	Baseline	0.530 (0.375 - 0.700)	4.66×10^{-2}	0.479 (0.391 - 0.721)	9.82×10^{-1}	2.26×10^{-2}
		Macenko	0.594 (0.431 - 0.791)	3.80×10^{-5}	0.591 (0.413 - 0.738)	1.33×10^{-5}	5.27×10^{-1}
		Reinhard	0.586 (0.348 - 0.764)	4.90×10^{-4}	0.572 (0.377 - 0.683)	2.78×10^{-5}	3.73×10^{-1}
		Grayscale	0.541 (0.314 - 0.741)	2.83×10^{-2}	0.523 (0.397 - 0.638)	4.78×10^{-2}	2.93×10^{-1}
		Normalized	0.578 (0.384 - 0.745)	1.90×10^{-4}	0.603 (0.413 - 0.746)	6.16×10^{-7}	8.65×10^{-1}
		Light	0.516 (0.365 - 0.672)	1.22×10^{-1}	0.524 (0.391 - 0.628)	3.15×10^{-2}	7.00×10^{-1}
		Heavy	0.540 (0.317 - 0.707)	1.34×10^{-2}	0.531 (0.396 - 0.622)	8.52×10^{-3}	3.91×10^{-1}
		Baseline	0.626 (0.547 - 0.739)	1.15×10^{-13}	0.607 (0.518 - 0.765)	3.65×10^{-9}	1.40×10^{-1}

	Immune Subtype	Macenko	0.646 (0.577 - 0.776)	2.15×10^{-14}	0.686 (0.491 - 0.828)	3.23×10^{-13}	1.00×10^{-0}
		Reinhard	0.621 (0.526 - 0.744)	2.98×10^{-13}	0.558 (0.449 - 0.685)	9.14×10^{-6}	5.12×10^{-5}
		Grayscale	0.680 (0.621 - 0.755)	9.07×10^{-19}	0.615 (0.466 - 0.734)	2.44×10^{-10}	2.11×10^{-5}
		Normalized	0.664 (0.589 - 0.748)	2.93×10^{-16}	0.618 (0.468 - 0.809)	3.26×10^{-7}	1.97×10^{-2}
		Light	0.639 (0.557 - 0.723)	1.05×10^{-16}	0.552 (0.416 - 0.675)	7.78×10^{-4}	2.59×10^{-6}
		Heavy	0.659 (0.586 - 0.736)	1.09×10^{-16}	0.610 (0.454 - 0.735)	6.19×10^{-8}	5.62×10^{-3}
	BRAF	Baseline	0.715 (0.535 - 0.896)	1.23×10^{-11}	0.699 (0.476 - 0.892)	7.90×10^{-9}	3.73×10^{-1}
		Macenko	0.743 (0.606 - 0.838)	1.03×10^{-17}	0.816 (0.674 - 0.886)	3.02×10^{-20}	1.00×10^{-0}
		Reinhard	0.779 (0.684 - 0.881)	5.82×10^{-21}	0.754 (0.526 - 0.851)	1.33×10^{-14}	1.39×10^{-1}
		Grayscale	0.712 (0.563 - 0.892)	6.57×10^{-12}	0.576 (0.285 - 0.790)	1.33×10^{-3}	2.23×10^{-5}
		Normalized	0.761 (0.626 - 0.926)	1.03×10^{-14}	0.753 (0.556 - 0.860)	1.28×10^{-15}	4.19×10^{-1}
		Light	0.742 (0.602 - 0.871)	1.28×10^{-14}	0.619 (0.358 - 0.729)	2.33×10^{-8}	1.49×10^{-6}
		Heavy	0.746 (0.593 - 0.889)	9.32×10^{-15}	0.555 (0.380 - 0.664)	3.35×10^{-4}	2.17×10^{-11}
	RNF43	Baseline	0.688 (0.472 - 0.825)	5.39×10^{-12}	0.494 (0.333 - 0.594)	7.18×10^{-1}	3.46×10^{-10}
		Macenko	0.701 (0.473 - 0.829)	2.29×10^{-13}	0.581 (0.327 - 0.741)	3.62×10^{-4}	2.11×10^{-5}
		Reinhard	0.726 (0.484 - 0.897)	5.19×10^{-13}	0.518 (0.249 - 0.780)	2.98×10^{-1}	5.90×10^{-8}
		Grayscale	0.736 (0.505 - 0.862)	3.16×10^{-14}	0.582 (0.434 - 0.752)	3.65×10^{-6}	9.58×10^{-9}
		Normalized	0.759 (0.564 - 0.885)	1.83×10^{-14}	0.737 (0.654 - 0.917)	1.28×10^{-15}	2.12×10^{-1}
		Light	0.724 (0.535 - 0.966)	1.06×10^{-12}	0.584 (0.438 - 0.867)	3.36×10^{-4}	8.35×10^{-6}
		Heavy	0.734 (0.541 - 0.870)	3.56×10^{-14}	0.611 (0.359 - 0.863)	5.49×10^{-4}	5.48×10^{-4}
	MSI Status	Baseline	0.616 (0.508 - 0.742)	1.40×10^{-8}	0.572 (0.361 - 0.843)	1.77×10^{-3}	7.99×10^{-2}
		Macenko	0.645 (0.528 - 0.789)	7.31×10^{-11}	0.641 (0.267 - 0.968)	1.31×10^{-4}	5.22×10^{-1}
		Reinhard	0.639 (0.556 - 0.754)	8.72×10^{-13}	0.640 (0.468 - 0.830)	3.71×10^{-6}	5.59×10^{-1}
		Grayscale	0.588 (0.398 - 0.744)	4.93×10^{-6}	0.601 (0.384 - 0.916)	1.03×10^{-3}	7.00×10^{-1}
		Normalized	0.633 (0.522 - 0.715)	4.35×10^{-13}	0.630 (0.382 - 0.926)	1.49×10^{-4}	5.22×10^{-1}
		Light	0.620 (0.472 - 0.728)	1.16×10^{-9}	0.579 (0.371 - 0.815)	1.22×10^{-3}	9.72×10^{-2}
		Heavy	0.610 (0.477 - 0.720)	1.16×10^{-8}	0.609 (0.372 - 0.852)	1.03×10^{-3}	5.29×10^{-1}
HNSC	Age	Baseline	0.526 (0.334 - 0.681)	6.56×10^{-2}	0.506 (0.394 - 0.609)	3.92×10^{-1}	2.36×10^{-1}
		Macenko	0.551 (0.444 - 0.678)	2.08×10^{-4}	0.520 (0.354 - 0.677)	1.98×10^{-1}	1.33×10^{-1}
		Reinhard	0.540 (0.424 - 0.631)	5.49×10^{-4}	0.534 (0.469 - 0.626)	1.68×10^{-3}	4.33×10^{-1}
		Grayscale	0.516 (0.419 - 0.632)	6.63×10^{-2}	0.516 (0.413 - 0.634)	1.52×10^{-1}	5.69×10^{-1}
		Normalized	0.556 (0.467 - 0.661)	3.27×10^{-6}	0.575 (0.488 - 0.663)	4.54×10^{-8}	9.34×10^{-1}
		Light	0.518 (0.432 - 0.610)	4.16×10^{-2}	0.501 (0.361 - 0.699)	6.18×10^{-1}	2.79×10^{-1}
		Heavy	0.522 (0.437 - 0.597)	9.03×10^{-3}	0.508 (0.406 - 0.629)	3.65×10^{-1}	2.70×10^{-1}
	Stage	Baseline	0.543 (0.450 - 0.608)	5.02×10^{-6}	0.489 (0.410 - 0.573)	9.79×10^{-1}	1.36×10^{-4}
		Macenko	0.569 (0.416 - 0.666)	3.71×10^{-6}	0.519 (0.436 - 0.596)	7.36×10^{-3}	1.76×10^{-3}
		Reinhard	0.519 (0.428 - 0.651)	3.01×10^{-2}	0.474 (0.377 - 0.553)	1.00×10^{-0}	3.47×10^{-3}
		Grayscale	0.524 (0.440 - 0.619)	1.72×10^{-3}	0.527 (0.424 - 0.704)	1.77×10^{-2}	6.47×10^{-1}
		Normalized	0.545 (0.445 - 0.659)	3.48×10^{-5}	0.499 (0.415 - 0.632)	6.83×10^{-1}	3.47×10^{-3}
		Light	0.548 (0.461 - 0.627)	1.88×10^{-6}	0.532 (0.443 - 0.725)	7.86×10^{-3}	1.97×10^{-1}
		Heavy	0.547 (0.468 - 0.647)	1.57×10^{-5}	0.537 (0.451 - 0.734)	2.20×10^{-3}	3.09×10^{-1}
	Gender	Baseline	0.560 (0.442 - 0.653)	2.21×10^{-7}	0.581 (0.459 - 0.675)	6.07×10^{-7}	9.34×10^{-1}
		Macenko	0.652 (0.528 - 0.792)	4.38×10^{-13}	0.590 (0.495 - 0.720)	2.72×10^{-7}	2.04×10^{-3}
		Reinhard	0.620 (0.492 - 0.730)	2.06×10^{-11}	0.619 (0.525 - 0.718)	2.44×10^{-11}	5.69×10^{-1}
		Grayscale	0.575 (0.447 - 0.735)	7.34×10^{-5}	0.544 (0.398 - 0.649)	1.37×10^{-3}	1.27×10^{-1}
		Normalized	0.622 (0.529 - 0.693)	1.71×10^{-14}	0.643 (0.553 - 0.701)	2.90×10^{-16}	9.73×10^{-1}
		Light	0.539 (0.413 - 0.646)	2.26×10^{-4}	0.543 (0.358 - 0.659)	2.38×10^{-3}	6.47×10^{-1}
		Heavy	0.560 (0.427 - 0.664)	5.70×10^{-6}	0.562 (0.405 - 0.694)	5.24×10^{-5}	6.12×10^{-1}
	Grade	Baseline	0.584 (0.452 - 0.704)	6.08×10^{-8}	0.555 (0.468 - 0.655)	1.75×10^{-5}	8.52×10^{-2}

		Macenko	0.665 (0.598 - 0.745)	1.68×10^{-17}	0.660 (0.580 - 0.741)	3.23×10^{-18}	3.88×10^{-1}
		Reinhard	0.598 (0.466 - 0.741)	2.44×10^{-8}	0.573 (0.454 - 0.704)	4.93×10^{-6}	1.48×10^{-1}
		Grayscale	0.587 (0.468 - 0.694)	9.53×10^{-8}	0.577 (0.476 - 0.678)	9.78×10^{-7}	3.53×10^{-1}
		Normalized	0.651 (0.525 - 0.752)	9.40×10^{-14}	0.629 (0.525 - 0.720)	5.40×10^{-12}	1.33×10^{-1}
		Light	0.622 (0.502 - 0.710)	3.76×10^{-13}	0.609 (0.485 - 0.724)	3.44×10^{-10}	2.60×10^{-1}
		Heavy	0.618 (0.491 - 0.715)	1.65×10^{-11}	0.589 (0.491 - 0.693)	7.61×10^{-10}	6.04×10^{-2}
	Ancestry	Baseline	0.720 (0.525 - 0.853)	2.21×10^{-12}	0.570 (0.303 - 0.829)	1.99×10^{-2}	3.78×10^{-4}
		Macenko	0.587 (0.342 - 0.869)	1.43×10^{-3}	0.541 (0.212 - 0.745)	1.17×10^{-1}	1.97×10^{-1}
		Reinhard	0.713 (0.451 - 0.866)	6.84×10^{-12}	0.649 (0.413 - 0.847)	4.03×10^{-6}	6.04×10^{-2}
		Grayscale	0.548 (0.327 - 0.705)	3.85×10^{-3}	0.454 (0.249 - 0.731)	1.00×10^{-0}	3.47×10^{-3}
		Normalized	0.579 (0.370 - 0.802)	1.70×10^{-3}	0.472 (0.229 - 0.731)	9.79×10^{-1}	5.17×10^{-3}
		Light	0.600 (0.349 - 0.828)	6.28×10^{-4}	0.483 (0.285 - 0.738)	8.90×10^{-1}	3.47×10^{-3}
		Heavy	0.553 (0.266 - 0.782)	3.05×10^{-2}	0.494 (0.287 - 0.804)	7.59×10^{-1}	1.11×10^{-1}
	HPV Status	Baseline	0.914 (0.800 - 0.970)	5.87×10^{-28}	0.846 (0.731 - 0.971)	3.13×10^{-17}	1.38×10^{-3}
		Macenko	0.902 (0.851 - 0.963)	4.23×10^{-29}	0.856 (0.658 - 0.976)	6.17×10^{-17}	2.42×10^{-2}
		Reinhard	0.929 (0.839 - 0.975)	2.17×10^{-29}	0.914 (0.810 - 0.989)	1.92×10^{-23}	1.97×10^{-1}
		Grayscale	0.908 (0.842 - 0.988)	2.77×10^{-29}	0.898 (0.739 - 0.990)	1.13×10^{-20}	3.09×10^{-1}
		Normalized	0.916 (0.855 - 0.976)	2.58×10^{-31}	0.911 (0.792 - 0.988)	9.57×10^{-23}	4.33×10^{-1}
		Light	0.931 (0.883 - 0.979)	1.03×10^{-32}	0.897 (0.785 - 0.980)	7.64×10^{-22}	1.74×10^{-2}
		Heavy	0.941 (0.911 - 0.976)	4.08×10^{-36}	0.905 (0.795 - 0.981)	1.92×10^{-23}	3.47×10^{-3}
	PFS	Baseline	0.614 (0.538 - 0.693)	5.54×10^{-13}	0.548 (0.431 - 0.728)	1.48×10^{-3}	6.35×10^{-4}
		Macenko	0.596 (0.360 - 0.738)	2.01×10^{-5}	0.417 (0.248 - 0.607)	1.00×10^{-0}	1.37×10^{-6}
		Reinhard	0.560 (0.453 - 0.640)	9.53×10^{-8}	0.483 (0.314 - 0.610)	9.79×10^{-1}	1.36×10^{-4}
		Grayscale	0.547 (0.413 - 0.702)	5.69×10^{-4}	0.512 (0.308 - 0.701)	3.24×10^{-1}	1.11×10^{-1}
		Normalized	0.532 (0.359 - 0.673)	3.26×10^{-2}	0.492 (0.360 - 0.643)	8.54×10^{-1}	8.10×10^{-2}
		Light	0.579 (0.418 - 0.698)	8.70×10^{-6}	0.463 (0.348 - 0.581)	1.00×10^{-0}	1.37×10^{-6}
		Heavy	0.560 (0.399 - 0.659)	4.50×10^{-5}	0.493 (0.383 - 0.633)	8.54×10^{-1}	1.19×10^{-3}
	RHOA	Baseline	0.733 (0.371 - 0.993)	5.96×10^{-5}	0.470 (0.347 - 0.857)	9.79×10^{-1}	1.36×10^{-4}
		Macenko	0.621 (0.303 - 0.986)	2.44×10^{-2}	0.598 (0.260 - 0.952)	4.08×10^{-2}	4.47×10^{-1}
		Reinhard	0.600 (0.238 - 0.993)	6.56×10^{-2}	0.315 (0.200 - 0.449)	1.00×10^{-0}	3.79×10^{-4}
		Grayscale	0.695 (0.236 - 0.986)	1.35×10^{-3}	0.562 (0.234 - 0.964)	2.15×10^{-1}	1.11×10^{-1}
		Normalized	0.664 (0.204 - 0.993)	8.58×10^{-3}	0.381 (0.134 - 0.926)	1.00×10^{-0}	2.42×10^{-3}
		Light	0.646 (0.187 - 0.986)	1.49×10^{-2}	0.472 (0.186 - 0.980)	8.41×10^{-1}	6.04×10^{-2}
		Heavy	0.629 (0.153 - 0.986)	3.14×10^{-2}	0.479 (0.163 - 0.946)	7.84×10^{-1}	1.11×10^{-1}
	TP53	Baseline	0.679 (0.557 - 0.840)	8.19×10^{-14}	0.691 (0.581 - 0.803)	6.09×10^{-14}	7.98×10^{-1}
		Macenko	0.683 (0.548 - 0.818)	2.42×10^{-14}	0.683 (0.581 - 0.810)	5.60×10^{-14}	5.69×10^{-1}
		Reinhard	0.716 (0.585 - 0.886)	1.67×10^{-13}	0.699 (0.609 - 0.850)	9.64×10^{-16}	2.66×10^{-1}
		Grayscale	0.712 (0.628 - 0.840)	1.35×10^{-16}	0.692 (0.556 - 0.793)	1.41×10^{-13}	2.10×10^{-1}
		Normalized	0.709 (0.603 - 0.887)	1.71×10^{-14}	0.675 (0.578 - 0.806)	5.27×10^{-15}	6.08×10^{-2}
		Light	0.717 (0.585 - 0.855)	1.52×10^{-15}	0.702 (0.567 - 0.823)	1.04×10^{-12}	2.86×10^{-1}
		Heavy	0.714 (0.590 - 0.888)	1.41×10^{-13}	0.694 (0.570 - 0.824)	5.52×10^{-13}	2.50×10^{-1}
	Immune Subtype	Baseline	0.651 (0.547 - 0.755)	1.49×10^{-14}	0.630 (0.443 - 0.750)	2.91×10^{-8}	2.17×10^{-1}
		Macenko	0.728 (0.637 - 0.819)	4.50×10^{-20}	0.740 (0.598 - 0.843)	4.59×10^{-16}	8.15×10^{-1}
		Reinhard	0.722 (0.617 - 0.847)	5.86×10^{-17}	0.678 (0.498 - 0.821)	9.48×10^{-11}	5.05×10^{-2}
		Grayscale	0.680 (0.549 - 0.775)	1.49×10^{-14}	0.649 (0.463 - 0.761)	3.17×10^{-11}	9.49×10^{-2}
		Normalized	0.749 (0.644 - 0.866)	3.52×10^{-16}	0.711 (0.576 - 0.778)	2.29×10^{-16}	5.05×10^{-2}
		Light	0.676 (0.547 - 0.795)	1.54×10^{-14}	0.652 (0.526 - 0.750)	1.64×10^{-13}	1.23×10^{-1}
		Heavy	0.717 (0.570 - 0.856)	1.89×10^{-15}	0.664 (0.508 - 0.764)	3.25×10^{-12}	8.38×10^{-3}
KIRC	Age	Baseline	0.561 (0.542 - 0.579)	2.93×10^{-6}	0.526 (0.487 - 0.565)	1.45×10^{-1}	1.98×10^{-2}

		Macenko	0.585 (0.536 - 0.635)	9.62×10^{-11}	0.507 (0.498 - 0.516)	9.14×10^{-3}	2.19×10^{-3}
		Reinhard	0.604 (0.574 - 0.633)	1.21×10^{-7}	0.533 (0.483 - 0.582)	4.05×10^{-1}	1.61×10^{-5}
		Grayscale	0.528 (0.491 - 0.565)	1.58×10^{-2}	0.522 (0.432 - 0.612)	7.52×10^{-2}	7.26×10^{-1}
		Normalized	0.581 (0.524 - 0.638)	4.80×10^{-7}	0.546 (0.494 - 0.598)	8.55×10^{-5}	5.35×10^{-1}
		Light	0.534 (0.477 - 0.592)	5.94×10^{-2}	0.531 (0.498 - 0.564)	7.76×10^{-3}	8.84×10^{-1}
		Heavy	0.525 (0.486 - 0.564)	2.23×10^{-2}	0.547 (0.478 - 0.616)	3.38×10^{-3}	9.35×10^{-1}
	Stage	Baseline	0.649 (0.576 - 0.730)	2.74×10^{-17}	0.582 (0.466 - 0.746)	7.40×10^{-6}	6.98×10^{-4}
		Macenko	0.685 (0.616 - 0.743)	3.31×10^{-20}	0.607 (0.541 - 0.710)	5.08×10^{-13}	6.99×10^{-9}
		Reinhard	0.645 (0.575 - 0.702)	5.26×10^{-18}	0.602 (0.453 - 0.749)	1.52×10^{-6}	3.00×10^{-2}
		Grayscale	0.646 (0.583 - 0.698)	4.40×10^{-19}	0.636 (0.497 - 0.756)	1.07×10^{-11}	3.09×10^{-1}
		Normalized	0.671 (0.586 - 0.745)	3.31×10^{-20}	0.648 (0.539 - 0.758)	1.62×10^{-13}	5.29×10^{-2}
		Light	0.644 (0.566 - 0.699)	5.08×10^{-19}	0.615 (0.465 - 0.747)	2.20×10^{-7}	9.44×10^{-2}
		Heavy	0.652 (0.573 - 0.701)	3.18×10^{-19}	0.620 (0.476 - 0.746)	1.23×10^{-7}	6.68×10^{-2}
	Gender	Baseline	0.656 (0.534 - 0.748)	1.29×10^{-14}	0.600 (0.497 - 0.708)	7.10×10^{-10}	1.02×10^{-3}
		Macenko	0.663 (0.568 - 0.724)	2.29×10^{-16}	0.610 (0.504 - 0.712)	5.19×10^{-12}	4.00×10^{-4}
		Reinhard	0.662 (0.584 - 0.750)	2.68×10^{-16}	0.577 (0.474 - 0.724)	2.76×10^{-6}	1.18×10^{-5}
		Grayscale	0.652 (0.536 - 0.733)	4.23×10^{-15}	0.630 (0.508 - 0.702)	6.88×10^{-13}	8.06×10^{-2}
		Normalized	0.633 (0.530 - 0.712)	9.80×10^{-14}	0.626 (0.542 - 0.718)	7.72×10^{-13}	3.93×10^{-1}
		Light	0.662 (0.580 - 0.757)	2.86×10^{-15}	0.623 (0.542 - 0.742)	1.12×10^{-12}	9.48×10^{-3}
		Heavy	0.676 (0.616 - 0.736)	1.02×10^{-18}	0.637 (0.553 - 0.720)	2.43×10^{-14}	2.19×10^{-3}
	Grade	Baseline	0.683 (0.597 - 0.829)	2.57×10^{-16}	0.665 (0.553 - 0.781)	2.43×10^{-14}	1.72×10^{-1}
		Macenko	0.739 (0.590 - 0.822)	3.31×10^{-20}	0.660 (0.539 - 0.764)	1.48×10^{-13}	4.07×10^{-6}
		Reinhard	0.687 (0.580 - 0.827)	1.29×10^{-16}	0.661 (0.507 - 0.806)	1.12×10^{-12}	8.74×10^{-2}
		Grayscale	0.702 (0.587 - 0.790)	5.27×10^{-18}	0.673 (0.526 - 0.787)	2.43×10^{-14}	5.09×10^{-2}
		Normalized	0.730 (0.585 - 0.800)	3.18×10^{-19}	0.702 (0.562 - 0.809)	5.42×10^{-15}	5.36×10^{-2}
		Light	0.717 (0.602 - 0.808)	1.62×10^{-18}	0.687 (0.568 - 0.802)	1.72×10^{-14}	5.09×10^{-2}
		Heavy	0.724 (0.618 - 0.802)	3.18×10^{-19}	0.696 (0.580 - 0.790)	8.48×10^{-17}	4.70×10^{-2}
	Ancestry	Baseline	0.759 (0.476 - 0.933)	3.49×10^{-11}	0.667 (0.135 - 0.903)	6.88×10^{-5}	5.09×10^{-2}
		Macenko	0.744 (0.518 - 0.910)	3.08×10^{-10}	0.615 (0.341 - 0.795)	8.03×10^{-6}	7.83×10^{-4}
		Reinhard	0.743 (0.541 - 0.915)	2.57×10^{-13}	0.559 (0.279 - 0.778)	3.01×10^{-2}	1.18×10^{-5}
		Grayscale	0.703 (0.466 - 0.883)	1.24×10^{-10}	0.512 (0.315 - 0.759)	3.27×10^{-1}	2.61×10^{-6}
		Normalized	0.776 (0.586 - 0.937)	1.83×10^{-14}	0.685 (0.443 - 0.929)	5.16×10^{-7}	1.49×10^{-2}
		Light	0.708 (0.493 - 0.879)	2.45×10^{-10}	0.633 (0.329 - 0.883)	1.44×10^{-5}	3.95×10^{-2}
		Heavy	0.715 (0.492 - 0.906)	4.84×10^{-11}	0.621 (0.383 - 0.864)	1.89×10^{-5}	7.89×10^{-3}
	PFS	Baseline	0.744 (0.613 - 0.875)	5.05×10^{-17}	0.680 (0.384 - 0.877)	2.01×10^{-6}	5.29×10^{-2}
		Macenko	0.756 (0.624 - 0.911)	2.74×10^{-17}	0.708 (0.492 - 0.850)	1.08×10^{-10}	5.09×10^{-2}
		Reinhard	0.739 (0.605 - 0.841)	3.18×10^{-19}	0.688 (0.444 - 0.866)	4.42×10^{-8}	5.36×10^{-2}
		Grayscale	0.712 (0.596 - 0.841)	8.05×10^{-16}	0.710 (0.536 - 0.867)	2.71×10^{-12}	5.35×10^{-1}
		Normalized	0.717 (0.608 - 0.806)	5.80×10^{-18}	0.690 (0.495 - 0.879)	3.25×10^{-9}	1.93×10^{-1}
		Light	0.715 (0.568 - 0.833)	6.69×10^{-17}	0.704 (0.532 - 0.897)	9.70×10^{-11}	4.06×10^{-1}
		Heavy	0.721 (0.573 - 0.833)	3.92×10^{-17}	0.724 (0.554 - 0.905)	8.46×10^{-12}	6.34×10^{-1}
	Immune Subtype	Baseline	0.643 (0.376 - 0.790)	9.98×10^{-7}	0.679 (0.383 - 0.952)	1.99×10^{-7}	8.85×10^{-1}
		Macenko	0.688 (0.482 - 0.867)	1.64×10^{-9}	0.674 (0.487 - 0.892)	9.00×10^{-9}	4.06×10^{-1}
		Reinhard	0.648 (0.359 - 0.874)	2.13×10^{-5}	0.742 (0.489 - 0.908)	1.73×10^{-12}	9.95×10^{-1}
		Grayscale	0.734 (0.188 - 0.891)	8.64×10^{-9}	0.723 (0.543 - 0.909)	2.71×10^{-12}	4.61×10^{-1}
		Normalized	0.744 (0.362 - 0.927)	1.19×10^{-9}	0.703 (0.446 - 0.880)	4.17×10^{-10}	1.72×10^{-1}
		Light	0.699 (0.456 - 0.909)	1.76×10^{-9}	0.717 (0.597 - 0.949)	8.57×10^{-12}	7.90×10^{-1}
		Heavy	0.768 (0.591 - 0.942)	5.78×10^{-14}	0.748 (0.582 - 0.931)	1.90×10^{-12}	3.14×10^{-1}
LUAD	Age	Baseline	0.528 (0.420 - 0.610)	3.15×10^{-3}	0.481 (0.297 - 0.606)	1.00×10^{-0}	4.78×10^{-3}

	Macenko	0.556 (0.455 - 0.670)	6.09×10^{-7}	0.519 (0.454 - 0.613)	2.65×10^{-2}	4.72×10^{-3}
	Reinhard	0.549 (0.418 - 0.649)	5.37×10^{-5}	0.588 (0.499 - 0.659)	1.76×10^{-9}	9.98×10^{-1}
	Grayscale	0.518 (0.374 - 0.597)	6.12×10^{-2}	0.554 (0.432 - 0.671)	2.21×10^{-4}	9.98×10^{-1}
	Normalized	0.505 (0.365 - 0.617)	3.81×10^{-1}	0.550 (0.458 - 0.623)	5.84×10^{-6}	9.98×10^{-1}
	Light	0.513 (0.426 - 0.598)	1.23×10^{-1}	0.504 (0.413 - 0.619)	4.61×10^{-1}	4.43×10^{-1}
	Heavy	0.500 (0.406 - 0.589)	5.44×10^{-1}	0.503 (0.429 - 0.616)	4.66×10^{-1}	7.87×10^{-1}
Stage	Baseline	0.599 (0.520 - 0.706)	2.46×10^{-11}	0.521 (0.416 - 0.637)	8.10×10^{-2}	7.76×10^{-6}
	Macenko	0.605 (0.528 - 0.682)	2.29×10^{-12}	0.538 (0.389 - 0.639)	5.27×10^{-3}	1.00×10^{-4}
	Reinhard	0.582 (0.508 - 0.661)	7.44×10^{-11}	0.501 (0.384 - 0.599)	6.13×10^{-1}	5.96×10^{-7}
	Grayscale	0.609 (0.495 - 0.677)	5.15×10^{-13}	0.528 (0.435 - 0.637)	1.11×10^{-2}	5.33×10^{-7}
	Normalized	0.627 (0.551 - 0.718)	1.42×10^{-14}	0.579 (0.471 - 0.663)	1.42×10^{-8}	4.36×10^{-4}
	Light	0.592 (0.477 - 0.694)	3.14×10^{-9}	0.499 (0.402 - 0.634)	7.03×10^{-1}	2.07×10^{-6}
	Heavy	0.593 (0.501 - 0.704)	7.64×10^{-10}	0.509 (0.402 - 0.633)	3.28×10^{-1}	4.42×10^{-6}
Gender	Baseline	0.551 (0.431 - 0.700)	1.41×10^{-3}	0.498 (0.406 - 0.591)	9.72×10^{-1}	8.39×10^{-3}
	Macenko	0.603 (0.499 - 0.711)	3.80×10^{-5}	0.572 (0.439 - 0.670)	9.53×10^{-3}	1.29×10^{-1}
	Reinhard	0.578 (0.425 - 0.718)	9.05×10^{-9}	0.519 (0.429 - 0.608)	1.06×10^{-1}	2.81×10^{-5}
	Grayscale	0.577 (0.458 - 0.718)	5.66×10^{-3}	0.512 (0.398 - 0.622)	1.10×10^{-3}	6.25×10^{-1}
	Normalized	0.587 (0.455 - 0.714)	9.01×10^{-12}	0.544 (0.420 - 0.609)	8.39×10^{-6}	3.86×10^{-5}
	Light	0.572 (0.467 - 0.673)	1.31×10^{-6}	0.560 (0.473 - 0.646)	3.43×10^{-5}	3.00×10^{-3}
	Heavy	0.601 (0.495 - 0.705)	2.48×10^{-8}	0.591 (0.518 - 0.677)	9.21×10^{-3}	1.56×10^{-5}
ALK Fusion	Baseline	0.637 (0.411 - 0.867)	2.06×10^{-5}	0.417 (0.213 - 0.630)	1.00×10^{-0}	2.80×10^{-7}
	Macenko	0.651 (0.518 - 0.833)	2.71×10^{-9}	0.483 (0.313 - 0.668)	9.66×10^{-1}	2.80×10^{-7}
	Reinhard	0.617 (0.424 - 0.947)	9.94×10^{-6}	0.408 (0.234 - 0.597)	1.00×10^{-0}	5.95×10^{-8}
	Grayscale	0.541 (0.254 - 0.727)	8.37×10^{-2}	0.401 (0.292 - 0.649)	1.00×10^{-0}	1.29×10^{-4}
	Normalized	0.547 (0.361 - 0.738)	1.24×10^{-2}	0.451 (0.189 - 0.695)	1.00×10^{-0}	4.78×10^{-3}
	Light	0.618 (0.384 - 0.973)	8.73×10^{-5}	0.428 (0.248 - 0.646)	1.00×10^{-0}	2.14×10^{-6}
	Heavy	0.599 (0.376 - 0.880)	2.79×10^{-4}	0.408 (0.246 - 0.583)	1.00×10^{-0}	3.59×10^{-7}
Ancestry	Baseline	0.678 (0.435 - 0.960)	5.00×10^{-8}	0.657 (0.476 - 0.872)	1.88×10^{-8}	4.20×10^{-1}
	Macenko	0.715 (0.580 - 0.954)	9.91×10^{-13}	0.648 (0.458 - 0.820)	1.91×10^{-9}	6.18×10^{-3}
	Reinhard	0.684 (0.453 - 0.951)	2.44×10^{-7}	0.607 (0.340 - 0.805)	4.63×10^{-5}	3.08×10^{-2}
	Grayscale	0.598 (0.396 - 0.783)	4.35×10^{-5}	0.537 (0.293 - 0.735)	1.01×10^{-1}	5.08×10^{-2}
	Normalized	0.625 (0.526 - 0.840)	3.31×10^{-9}	0.517 (0.234 - 0.779)	4.03×10^{-1}	2.85×10^{-3}
	Light	0.642 (0.447 - 0.924)	2.62×10^{-7}	0.556 (0.297 - 0.739)	4.50×10^{-3}	3.81×10^{-3}
	Heavy	0.630 (0.494 - 0.902)	3.17×10^{-7}	0.522 (0.286 - 0.704)	2.28×10^{-1}	7.39×10^{-4}
PFS	Baseline	0.525 (0.361 - 0.620)	5.53×10^{-2}	0.523 (0.344 - 0.692)	1.56×10^{-1}	6.81×10^{-1}
	Macenko	0.535 (0.371 - 0.634)	2.41×10^{-3}	0.467 (0.360 - 0.574)	1.00×10^{-0}	2.44×10^{-4}
	Reinhard	0.488 (0.365 - 0.620)	9.03×10^{-1}	0.495 (0.322 - 0.633)	7.54×10^{-1}	8.24×10^{-1}
	Grayscale	0.473 (0.375 - 0.584)	9.99×10^{-1}	0.471 (0.304 - 0.633)	1.00×10^{-0}	6.81×10^{-1}
	Normalized	0.455 (0.300 - 0.665)	9.99×10^{-1}	0.486 (0.347 - 0.664)	9.66×10^{-1}	9.98×10^{-1}
	Light	0.517 (0.391 - 0.638)	7.52×10^{-2}	0.514 (0.380 - 0.630)	2.15×10^{-1}	6.63×10^{-1}
	Heavy	0.464 (0.364 - 0.596)	9.99×10^{-1}	0.475 (0.318 - 0.582)	1.00×10^{-0}	9.12×10^{-1}
STK11	Baseline	0.645 (0.528 - 0.804)	1.83×10^{-10}	0.660 (0.468 - 0.834)	3.00×10^{-8}	9.12×10^{-1}
	Macenko	0.708 (0.575 - 0.858)	1.36×10^{-13}	0.667 (0.448 - 0.850)	3.52×10^{-9}	7.83×10^{-2}
	Reinhard	0.663 (0.531 - 0.772)	3.66×10^{-12}	0.725 (0.495 - 0.895)	3.01×10^{-10}	9.98×10^{-1}
	Grayscale	0.625 (0.462 - 0.780)	5.11×10^{-8}	0.622 (0.429 - 0.840)	5.92×10^{-5}	6.81×10^{-1}
	Normalized	0.654 (0.548 - 0.750)	6.81×10^{-13}	0.653 (0.523 - 0.797)	2.59×10^{-10}	7.01×10^{-1}
	Light	0.624 (0.507 - 0.735)	4.47×10^{-10}	0.649 (0.347 - 0.865)	1.11×10^{-5}	9.57×10^{-1}
	Heavy	0.649 (0.515 - 0.766)	2.92×10^{-11}	0.657 (0.440 - 0.889)	3.75×10^{-6}	7.87×10^{-1}
TP53	Baseline	0.667 (0.547 - 0.778)	1.36×10^{-13}	0.709 (0.584 - 0.794)	4.19×10^{-16}	9.98×10^{-1}

		Macenko	0.709 (0.483 - 0.841)	3.09×10^{-13}	0.712 (0.615 - 0.786)	1.00×10^{-18}	7.85×10^{-1}
		Reinhard	0.701 (0.549 - 0.817)	2.50×10^{-15}	0.719 (0.617 - 0.810)	2.93×10^{-18}	9.98×10^{-1}
		Grayscale	0.679 (0.544 - 0.802)	8.58×10^{-15}	0.710 (0.640 - 0.798)	2.13×10^{-18}	9.98×10^{-1}
		Normalized	0.702 (0.576 - 0.795)	8.58×10^{-15}	0.729 (0.642 - 0.800)	3.18×10^{-21}	9.98×10^{-1}
		Light	0.671 (0.567 - 0.782)	1.42×10^{-14}	0.707 (0.632 - 0.774)	1.82×10^{-20}	9.98×10^{-1}
		Heavy	0.669 (0.562 - 0.792)	9.51×10^{-14}	0.705 (0.634 - 0.777)	1.36×10^{-20}	9.98×10^{-1}
	Immune Subtype	Baseline	0.598 (0.527 - 0.680)	7.01×10^{-12}	0.529 (0.426 - 0.616)	3.73×10^{-3}	2.67×10^{-6}
		Macenko	0.646 (0.549 - 0.769)	4.76×10^{-13}	0.600 (0.524 - 0.691)	1.37×10^{-10}	3.66×10^{-3}
		Reinhard	0.650 (0.569 - 0.765)	1.42×10^{-14}	0.612 (0.517 - 0.714)	1.14×10^{-12}	4.25×10^{-3}
		Grayscale	0.644 (0.552 - 0.780)	6.81×10^{-13}	0.645 (0.559 - 0.726)	1.86×10^{-16}	7.21×10^{-1}
		Normalized	0.661 (0.552 - 0.804)	4.89×10^{-12}	0.625 (0.540 - 0.766)	1.14×10^{-12}	3.02×10^{-2}
		Light	0.647 (0.552 - 0.745)	1.02×10^{-13}	0.615 (0.518 - 0.710)	2.06×10^{-11}	2.48×10^{-2}
		Heavy	0.664 (0.582 - 0.760)	1.52×10^{-15}	0.627 (0.501 - 0.732)	8.50×10^{-11}	1.36×10^{-2}
LUSC	Age	Baseline	0.532 (0.404 - 0.680)	1.07×10^{-2}	0.476 (0.306 - 0.713)	1.00×10^{-0}	1.21×10^{-2}
		Macenko	0.475 (0.333 - 0.641)	9.47×10^{-1}	0.459 (0.344 - 0.565)	1.00×10^{-0}	2.21×10^{-1}
		Reinhard	0.542 (0.361 - 0.674)	6.09×10^{-3}	0.485 (0.339 - 0.638)	1.00×10^{-0}	6.71×10^{-3}
		Grayscale	0.470 (0.229 - 0.652)	9.47×10^{-1}	0.495 (0.344 - 0.625)	1.00×10^{-0}	8.67×10^{-1}
		Normalized	0.515 (0.267 - 0.757)	2.33×10^{-1}	0.497 (0.327 - 0.640)	1.00×10^{-0}	2.35×10^{-1}
		Light	0.521 (0.357 - 0.711)	1.00×10^{-1}	0.469 (0.348 - 0.664)	1.00×10^{-0}	1.38×10^{-2}
		Heavy	0.506 (0.373 - 0.697)	3.58×10^{-1}	0.480 (0.323 - 0.678)	1.00×10^{-0}	1.29×10^{-1}
	Stage	Baseline	0.537 (0.440 - 0.633)	1.54×10^{-3}	0.466 (0.348 - 0.599)	1.00×10^{-0}	2.64×10^{-5}
		Macenko	0.553 (0.453 - 0.652)	1.57×10^{-4}	0.476 (0.395 - 0.541)	1.00×10^{-0}	1.72×10^{-6}
		Reinhard	0.573 (0.474 - 0.678)	1.17×10^{-5}	0.475 (0.411 - 0.548)	1.00×10^{-0}	1.65×10^{-7}
		Grayscale	0.557 (0.449 - 0.675)	2.12×10^{-4}	0.471 (0.368 - 0.606)	1.00×10^{-0}	1.56×10^{-5}
		Normalized	0.598 (0.475 - 0.699)	1.93×10^{-7}	0.557 (0.433 - 0.729)	2.27×10^{-3}	2.80×10^{-2}
		Light	0.555 (0.470 - 0.694)	1.83×10^{-4}	0.486 (0.364 - 0.586)	1.00×10^{-0}	4.60×10^{-4}
		Heavy	0.593 (0.490 - 0.722)	1.85×10^{-7}	0.493 (0.368 - 0.609)	1.00×10^{-0}	5.68×10^{-6}
	Gender	Baseline	0.544 (0.385 - 0.659)	1.41×10^{-3}	0.502 (0.418 - 0.579)	9.72×10^{-1}	8.39×10^{-3}
		Macenko	0.552 (0.423 - 0.672)	3.80×10^{-5}	0.534 (0.396 - 0.690)	9.53×10^{-3}	1.29×10^{-1}
		Reinhard	0.611 (0.458 - 0.715)	9.05×10^{-9}	0.526 (0.387 - 0.647)	1.06×10^{-1}	2.81×10^{-5}
		Grayscale	0.540 (0.367 - 0.669)	5.66×10^{-3}	0.545 (0.417 - 0.686)	1.10×10^{-3}	6.25×10^{-1}
		Normalized	0.641 (0.484 - 0.742)	9.01×10^{-12}	0.570 (0.467 - 0.738)	8.39×10^{-6}	3.86×10^{-5}
		Light	0.593 (0.426 - 0.700)	1.31×10^{-6}	0.543 (0.456 - 0.610)	3.43×10^{-5}	3.00×10^{-3}
		Heavy	0.597 (0.441 - 0.672)	2.48×10^{-8}	0.527 (0.440 - 0.640)	9.21×10^{-3}	1.56×10^{-5}
	ALK Fusion	Baseline	0.678 (0.276 - 0.989)	3.37×10^{-4}	0.404 (0.122 - 0.875)	1.00×10^{-0}	9.29×10^{-5}
		Macenko	0.673 (0.298 - 0.977)	4.38×10^{-4}	0.610 (0.180 - 0.905)	9.53×10^{-3}	1.48×10^{-1}
		Reinhard	0.757 (0.456 - 0.977)	1.36×10^{-9}	0.299 (0.006 - 0.773)	1.00×10^{-0}	2.27×10^{-10}
		Grayscale	0.595 (0.017 - 0.920)	4.14×10^{-2}	0.291 (0.110 - 0.432)	1.00×10^{-0}	1.26×10^{-6}
		Normalized	0.748 (0.421 - 1.000)	2.53×10^{-9}	0.322 (0.059 - 0.875)	1.00×10^{-0}	4.06×10^{-9}
		Light	0.661 (0.234 - 0.977)	2.88×10^{-4}	0.358 (0.141 - 0.841)	1.00×10^{-0}	1.64×10^{-6}
		Heavy	0.653 (0.200 - 0.977)	8.98×10^{-5}	0.308 (0.090 - 0.591)	1.00×10^{-0}	2.21×10^{-10}
	Ancestry	Baseline	0.789 (0.472 - 0.983)	1.68×10^{-10}	0.504 (0.245 - 0.802)	9.78×10^{-1}	1.26×10^{-9}
		Macenko	0.713 (0.474 - 0.967)	4.53×10^{-10}	0.455 (0.257 - 0.747)	1.00×10^{-0}	1.11×10^{-10}
		Reinhard	0.721 (0.390 - 0.990)	1.73×10^{-7}	0.479 (0.260 - 0.703)	1.00×10^{-0}	7.11×10^{-8}
		Grayscale	0.596 (0.340 - 0.753)	1.43×10^{-5}	0.458 (0.162 - 0.757)	1.00×10^{-0}	3.83×10^{-4}
		Normalized	0.644 (0.370 - 0.833)	1.19×10^{-6}	0.398 (0.249 - 0.602)	1.00×10^{-0}	1.32×10^{-10}
		Light	0.570 (0.279 - 0.778)	4.42×10^{-3}	0.427 (0.298 - 0.570)	1.00×10^{-0}	6.95×10^{-6}
		Heavy	0.581 (0.297 - 0.794)	1.35×10^{-3}	0.391 (0.217 - 0.619)	1.00×10^{-0}	2.31×10^{-7}
	PFS	Baseline	0.589 (0.411 - 0.726)	8.00×10^{-7}	0.485 (0.346 - 0.629)	1.00×10^{-0}	5.50×10^{-7}

	Macenko	0.578 (0.438 - 0.781)	8.36×10^{-6}	0.537 (0.376 - 0.655)	1.84×10^{-2}	2.40×10^{-2}
	Reinhard	0.585 (0.464 - 0.795)	9.13×10^{-6}	0.530 (0.376 - 0.637)	5.32×10^{-2}	7.35×10^{-3}
	Grayscale	0.597 (0.410 - 0.750)	1.53×10^{-5}	0.549 (0.309 - 0.706)	2.79×10^{-2}	4.26×10^{-2}
	Normalized	0.627 (0.406 - 0.824)	5.48×10^{-7}	0.574 (0.393 - 0.710)	6.45×10^{-4}	2.40×10^{-2}
	Light	0.560 (0.433 - 0.697)	2.18×10^{-4}	0.461 (0.266 - 0.550)	1.00×10^{-0}	1.47×10^{-5}
	Heavy	0.605 (0.448 - 0.800)	6.90×10^{-7}	0.485 (0.253 - 0.630)	1.00×10^{-0}	2.20×10^{-5}
PIK3R1	Baseline	0.614 (0.288 - 1.000)	2.51×10^{-2}	0.386 (0.245 - 0.691)	1.00×10^{-0}	3.19×10^{-4}
	Macenko	0.655 (0.141 - 1.000)	2.39×10^{-3}	0.294 (0.123 - 0.625)	1.00×10^{-0}	1.18×10^{-7}
	Reinhard	0.733 (0.225 - 1.000)	1.01×10^{-6}	0.371 (0.033 - 0.849)	1.00×10^{-0}	2.86×10^{-6}
	Grayscale	0.790 (0.265 - 1.000)	1.94×10^{-8}	0.651 (0.066 - 0.954)	8.02×10^{-3}	1.38×10^{-2}
	Normalized	0.730 (0.099 - 1.000)	9.13×10^{-6}	0.516 (0.079 - 0.901)	8.70×10^{-1}	7.60×10^{-4}
	Light	0.670 (0.089 - 1.000)	4.72×10^{-4}	0.577 (0.066 - 0.947)	1.20×10^{-1}	8.08×10^{-2}
	Heavy	0.735 (0.053 - 1.000)	5.96×10^{-6}	0.588 (0.020 - 0.941)	1.18×10^{-1}	1.53×10^{-2}
Immune Subtype	Baseline	0.605 (0.509 - 0.672)	8.71×10^{-12}	0.505 (0.425 - 0.590)	6.78×10^{-1}	1.32×10^{-10}
	Macenko	0.620 (0.503 - 0.727)	7.39×10^{-12}	0.568 (0.468 - 0.701)	3.43×10^{-5}	8.97×10^{-4}
	Reinhard	0.628 (0.500 - 0.694)	7.39×10^{-12}	0.538 (0.426 - 0.675)	2.92×10^{-3}	1.49×10^{-7}
	Grayscale	0.616 (0.505 - 0.684)	7.39×10^{-12}	0.585 (0.460 - 0.662)	4.92×10^{-7}	2.27×10^{-2}
	Normalized	0.666 (0.551 - 0.766)	1.59×10^{-13}	0.600 (0.509 - 0.660)	5.40×10^{-11}	8.05×10^{-6}
	Light	0.617 (0.489 - 0.743)	3.28×10^{-9}	0.514 (0.424 - 0.631)	1.97×10^{-1}	1.18×10^{-7}
	Heavy	0.614 (0.480 - 0.688)	7.80×10^{-11}	0.540 (0.447 - 0.646)	6.23×10^{-5}	5.50×10^{-7}

Standard refers to grayscale standard normalization, Light refers to light hue, saturation, and value augmentation, and Heavy refers to heavy hue, saturation, and value augmentation. P-values listed are false discovery corrected p-values for one sided t-test comparison to AUROC of 0.50, with significance indicating a better than random performance at predicting the outcome of interest, as well as for a one sided t-test comparing standard to preserved site AUROC, with significance indicating a significant decline in AUROC with site preserved cross validation. P-values listed are Benjamini-Hochberg corrected for a false discovery rate of 0.05, with (*) indicating p-values maintain significance with correction (calculated per cancer subtype).

Supplementary Table 8. Summary Statistics for Supplementary Table 6.

Stain Normalization	Average Difference in AUROC (Standard vs Preserved Site Cross Validation)	Features Predictable with Standard Cross Validation (%)	Predictable Features with Decrease in AUROC with Preserved Site Validation (%)	Features No Longer Predictable with Preserved site Validation (%)
Baseline	0.069	56 (96.6)	51 (91.1)	20 (35.7)
Macenko	0.053	57 (98.3)	50 (87.7)	10 (17.5)
Reinhard	0.072	56 (96.6)	48 (85.7)	15 (26.8)
Grayscale	0.047	52 (89.7)	44 (84.6)	12 (23.1)
Grayscale Normalized	0.056	55 (94.8)	49 (89.1)	11 (20.0)
Light HSV Augmentation	0.053	52 (89.7)	46 (88.5)	13 (25.0)
Heavy HSV Augmentation	0.056	55 (94.8)	49 (89.1)	14 (25.5)

Supplementary Table 9. False Positive Rate for Prediction of African Ancestry, Balanced by Race versus Preserved Sites.

Slide Adjustment Method	False Positive AFR Balanced by Race (%)	False Positive AFR Preserved Sites (%)	Chi-squared statistic	p-value
Baseline	80.6	0.1	2964.3	$< 1 \times 10^{--300}$
Macenko	37.4	3.4	787.3	4.45×10^{-173}
Reinhard	48.1	3.4	1148.9	1.80×10^{-251}
Grayscale	36.7	14.1	296.6	$1.82 \times 10^{--66}$
Grayscale Normalized	38.8	9.3	521.1	$2.89 \times 10^{--115}$
Light HSV Augmentation	49.7	6.0	1045.4	$4.26 \times 10^{--229}$
Heavy HSV Augmentation	67.8	14.6	1289.7	$6.94 \times 10^{--282}$

False positive prediction of European ancestry and African ancestry for patients within the University of Chicago dataset (measured at the tile level, n = 2,206 tiles from 20 patients, 17 with African ancestry, 3 with European ancestry) for models trained with standard and preserved site cross validation. Slide normalization techniques improve the false positive rate, but not to the same level as isolating sites to either validation or testing sets. P-values are for a chi-squared test comparing the number of true / false positive predictions for African Ancestry with the two methods of cross validation. P-values listed are Benjamini-Hochberg corrected for a false discovery rate of 0.05, with (*) indicating p-values maintain significance with correction.
