# Supplementary material for: The impact of site-specific digital histology signatures on deep learning model accuracy and bias
Source: Nat Commun. 2021 Jul 20;12:4423. doi: 10.1038/s41467-021-24698-1 (PMC8292530; doi:10.1038/s41467-021-24698-1)
Supplement: Supplementary file 2 — Reporting Summary [file 41467_2021_24698_MOESM2_ESM.pdf]

nature research

Corresponding author(s): Alexander Pearson, Robert Grossman

Last updated by author(s): 4/26/2021

Reporting Summary

Nature Research wishes to improve the reproducibility of the work that we publish. This form provides structure for consistency and transparency in reporting. For further information on Nature Research policies, see our Editorial Policies and the Editorial Policy Checklist.

Statistics

For all statistical analyses, confirm that the following items are present in the figure legend, table legend, main text, or Methods section.

n/a	Confirmed	
☐	☒	The exact sample size (n) for each experimental group/condition, given as a discrete number and unit of measurement
☐	☒	A statement on whether measurements were taken from distinct samples or whether the same sample was measured repeatedly
☐	☒	The statistical test(s) used AND whether they are one- or two-sided<br>*Only common tests should be described solely by name; describe more complex techniques in the Methods section.*
☐	☒	A description of all covariates tested
☐	☒	A description of any assumptions or corrections, such as tests of normality and adjustment for multiple comparisons
☐	☒	A full description of the statistical parameters including central tendency (e.g. means) or other basic estimates (e.g. regression coefficient) AND variation (e.g. standard deviation) or associated estimates of uncertainty (e.g. confidence intervals)
☐	☒	For null hypothesis testing, the test statistic (e.g. F, t, r) with confidence intervals, effect sizes, degrees of freedom and P value noted<br>*Give P values as exact values whenever suitable.*
☒	☐	For Bayesian analysis, information on the choice of priors and Markov chain Monte Carlo settings
☒	☐	For hierarchical and complex designs, identification of the appropriate level for tests and full reporting of outcomes
☒	☐	Estimates of effect sizes (e.g. Cohen's d, Pearson's r), indicating how they were calculated

Our web collection on statistics for biologists contains articles on many of the points above.

Software and code

Policy information about availability of computer code

Data collection	No software was used for data collection
Data analysis	Slide processing was performed with using QuPath version 0.12. Image analysis was performed using the scikit-image 0.18.0. Deep learning models were created with the Xception architecture included in TensorFlow 2.3.0. Macenko and Reinhard stain normalization was performed with the StainTools v2.1.3 package. Custom software used for crossfold generation is provided on GitHub at https://github.com/fmhoward/PreservedSiteCV. All data analysis was performed using Python 3.8.

For manuscripts utilizing custom algorithms or software that are central to the research but not yet described in published literature, software must be made available to editors and reviewers. We strongly encourage code deposition in a community repository (e.g. GitHub). See the Nature Research guidelines for submitting code & software for further information.

Data

Policy information about availability of data

All manuscripts must include a data availability statement. This statement should provide the following information, where applicable:

- Accession codes, unique identifiers, or web links for publicly available datasets
- A list of figures that have associated raw data
- A description of any restrictions on data availability

Data from TCGA including digital histology and the clinical and genetic annotations used are available from https://portal.gdc.cancer.gov/ and https://cbioportal.org. Annotations for immune subtype are available from the published work of Thorsson et al33, and annotations for genomic ancestry were obtained from Carrot-Zhang et al41. Annotations for driver mutations are available from https://github.com/jnkather/DeepHistology. Source data are provided with this paper, which highlight all the relevant findings of this work including the range and summary statistics of bootstrapped AUROCs generated. Any additional data is available on request. ImageNet pretraining weights for deep learning models are included as part of the TensorFlow 2.3.0 package.

Field-specific reporting

Please select the one below that is the best fit for your research. If you are not sure, read the appropriate sections before making your selection.

☒ Life sciences ☐ Behavioural & social sciences ☐ Ecological, evolutionary & environmental sciences

For a reference copy of the document with all sections, see nature.com/documents/nr-reporting-summary-flat.pdf

Life sciences study design

All studies must disclose on these points even when the disclosure is negative.

Sample size	No sample size calculation was performed - sample sizes were based on the number of patients available in TCGA, and we consider these reasonable as this dataset has been used for internal validation for clinical and genomic outcomes in a variety of other studies.
Data exclusions	Patients in TCGA without digital histology available are excluded as histology is central to our analysis.
Replication	Key findings such as the data splits generated by the preserved site cross validation algorithm and the AUROCs predicted by the trained deep learning models were assessed in duplicate with identical results. We have confirmed this in the statistics and reproducibility section. We have also provided the exact splits of data used for each experiment in our github repository to aid in reproducibility.
Randomization	With standard cross validation, patients were randomly allocated into three groups for evaluation while ensuring optimal stratification by the outcome of interest. With preserved site cross validation, sites were assigned to ensure adequate stratification by the outcome of interest, but the allocation was otherwise nondeterministic. As we describe in the limitations of the study, there may be confounders present - and indeed not all relevant confounders are documented within the TCGA dataset, such as grade for breast cancer which is correlated with ER status. However, the point of this manuscript is that if such covariates are only correlated with the outcome of interest in one tissue submitting site in TCGA, models trained to learn that association are not likely to generalize, as we discuss.
Blinding	Blinding was not performed due to the retrospective nature of this study.

Reporting for specific materials, systems and methods

We require information from authors about some types of materials, experimental systems and methods used in many studies. Here, indicate whether each material, system or method listed is relevant to your study. If you are not sure if a list item applies to your research, read the appropriate section before selecting a response.

Materials & experimental systems

n/a	Involved in the study
☒	☐ Antibodies
☒	☐ Eukaryotic cell lines
☒	☐ Palaeontology and archaeology
☒	☐ Animals and other organisms
☐	☒ Human research participants
☒	☐ Clinical data
☒	☐ Dual use research of concern

Methods

n/a	Involved in the study
☒	☐ ChIP-seq
☒	☐ Flow cytometry
☒	☐ MRI-based neuroimaging

Human research participants

Policy information about studies involving human research participants

Population characteristics	All human subject data was obtained from The Cancer Genome Atlas. Patients of several cancer subtypes were included; the demographic information of these subsets is described in Supplemental Table 4.
Recruitment	Participant recruitment to TCGA has previously been described for each of the datasets used in this study, with citations in the methods section of this text. A number of biases exist in this dataset, including the relative under-representation of minority patients. Describing the bias present in recruitment that differs between submitting sites is one of the central finding of our submitted work.
Ethics oversight	Ethics oversight of the TCGA is described at https://www.cancer.gov/about-nci/organization/ccg/research/structural-genomics/tcga/history/policies

Note that full information on the approval of the study protocol must also be provided in the manuscript.
